# Rhenium(i) trinuclear rings as highly efficient redox photosensitizers for photocatalytic CO_2_ reduction†
†Electronic supplementary information (ESI) available: Franck–Condon analysis; photochemical one-electron-reduced species formation and characterisation; photophysical, electrochemical and quenching properties of **R(4·5)** in DMA; photophysical and electrochemical properties of the catalysts; photocatalytic CO_2_ reduction experiments and additional data. See DOI: 10.1039/c6sc01913g



**DOI:** 10.1039/c6sc01913g

**Published:** 2016-07-05

**Authors:** Jana Rohacova, Osamu Ishitani

**Affiliations:** a Department of Chemistry , Graduate School of Science and Engineering , Tokyo Institute of Technology , 2-12-1-NE-1 Ookayama , Meguro-ku , Tokyo 152-8550 , Japan . Email: ishitani@chem.titech.ac.jp

## Abstract

We developed new cyclic Re(i)-based trinuclear redox photosensitizers with both high oxidation power in the excited state and strong reduction power in the reduced form. These excellent properties were achieved by introducing electron-donating groups on the diimine ligand of the Re(i) metal centre and by connecting each Re(i) unit with polyphenyl–bisphosphine bridging ligands. These Re-rings were applied to homogenous visible light-driven photocatalytic CO_2_ reduction in conjunction with various mononuclear catalysts, such as Re(i), Ru(ii) and Mn(i) metal complexes, employing a relatively weak sacrificial electron donor, triethanolamine. Each system showed good product selectivity (CO or HCOOH) and an excellent quantum yield of product formation *Φ*_CO_ = 0.60 to 0.74 using *fac*-[Re^I^(bpy)(CO)_3_(CH_3_CN)]^+^, *Φ*_HCOOH_ = 0.58 using *trans*(Cl)–Ru^II^(dtbb)(CO)_2_Cl_2_ and *Φ*_HCOOH_ = 0.48 using a *fac*-[Mn^I^(dtbb)(CO)_3_(CH_3_CN)]^+^ catalyst. The high photocatalytic efficiencies for CO_2_ reduction are attributed to efficient reductive quenching of the Re-ring by triethanolamine and fast electron transfer from the generated one-electron-reduced species of the ring to the catalyst.

## Abstract 2

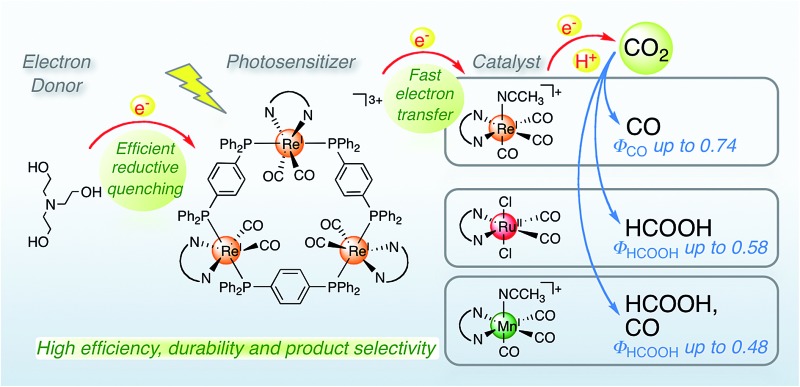
Trinuclear Re(i)-rings were applied as redox photosensitizers in visible light-driven CO_2_ reduction in tandem with various catalysts, *i.e.*, Re(i)-, Ru(ii)- and Mn(i)-diimine metal complex. The quantum yields for the Ru(ii) and Mn(i) catalysts were the among highest reported.

## Introduction

Redox photosensitizers (PSs) have been widely used in various photocatalytic reactions such as for organic synthesis, dye-sensitized solar cells, photoinduced H_2_ or O_2_ production from water and reduction of CO_2_.^1–8^ The first step of the photosensitization is the photoexcitation of the PS, followed by a reductive or oxidative quenching reaction with a substrate or semiconductor particles and electrodes. The produced one-electron-reduced or one-electron-oxidized species (OERS or OEOS, respectively) donates an electron or hole, respectively, to another substrate in the final process of the photosensitization. Therefore, PSs are required to have the following properties: (1) stability of the excited state, (2) stability of the OERS and/or OEOS, (3) strong oxidation and/or reduction power in the excited state and (4) strong reduction or oxidation power of the OERS or OEOS.

Some transition-metal complexes are frequently used as PSs not only because they fulfil the aforementioned requirements but also because they have a strong absorption in the visible region, which is an important feature for solar energy conversion. Most reported PSs are mononuclear metal complexes, and their types have been limited mostly to Ru(ii)-diimine and cyclometalated-Ir(iii) complexes and their derivatives. Although some metal-porphyrins and metal-phthalocyanines as well as Pt(ii)-, Os(ii)-, Re(i)- and Fe(ii)-diimine complexes have also been investigated as PSs, they are used only in limited types of reactions.^8–14^ On the contrary, the possibilities of using multi-nuclear metal complexes as PSs have only been scarcely investigated to date.^15^

We recently reported the photochemical synthesis of ring-shaped multinuclear Re(i) complexes with *cis*, *trans*-[Re(bpy)(CO)_2_(P–P)_2_]^+^ (bpy = 2,2′-bipyridine, P–P = PPh_2_–(C_*n*_H_*m*_)–PPh_2_) as repeating units.^16–18^ They exhibited outstanding photophysical and electrochemical properties, such as high emission quantum yields, along with long lifetimes of the ^3^MLCT excited states, even in solution at room temperature, and stability in the excited state and stronger oxidation power in the excited state compared to the corresponding mononuclear Re complex. They can also photochemically accumulate multiple electrons in one molecule. In the first report on these Re-rings, we also briefly introduced the idea that the Re-ring could be used as an extremely efficient PS for photocatalytic CO_2_ reduction in tandem with a Re(i) catalyst under visible light irradiation.

**Chart 1:**
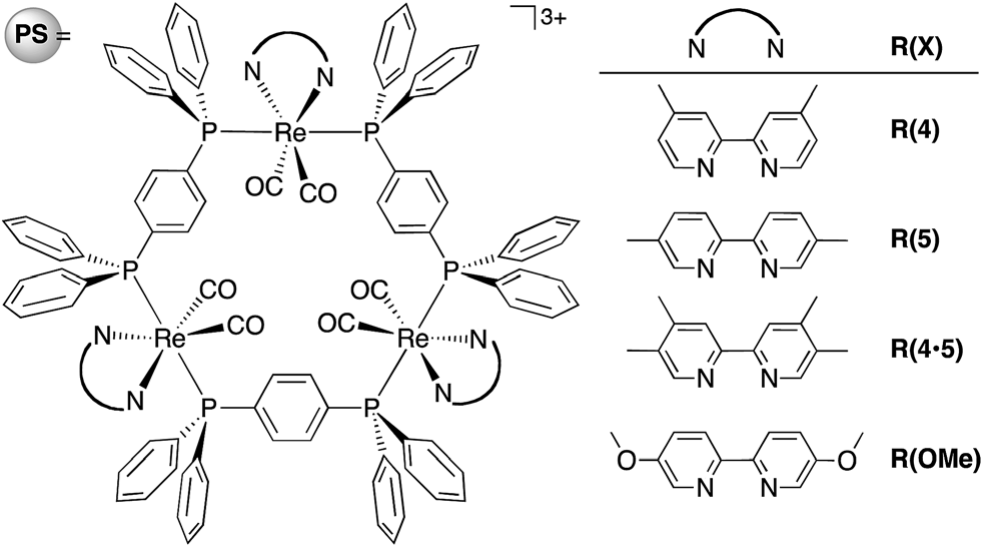
Structure and abbreviations of the trinuclear Re(i) rings **R(X)**. All complexes were synthesized as PF_6_^–^ salts.

**Chart 2:**
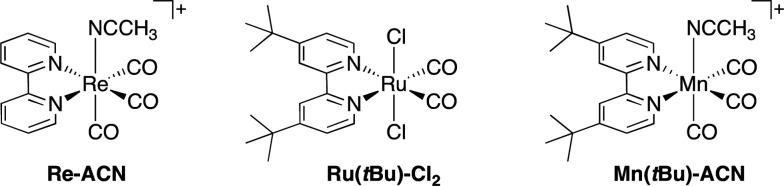
Structures and abbreviations of the Re(i), Ru(ii) and Mn(i) catalysts. Re(i) and Mn(i) complexes were synthesized as PF_6_^–^ salts.

Herein, we report the potentialities of these Re-rings as PSs in detail. Newly designed and synthesized trinuclear Re-rings, **R(X)**, where each Re(i) unit is connected with *p*-bis(diphenylphosphino)benzene (Chart 1), were applied to photocatalytic CO_2_ reduction with three kinds of typical catalysts, namely *fac*-[Re(bpy)(CO)_3_(CH_3_CN)]^+^, *trans*(Cl)–Ru(dtbb)(CO)_2_Cl_2_ and *fac*-[Mn(dtbb)(CO)_3_(CH_3_CN)]^+^ (dtbb = 4,4′-di-*tert*-butyl-2,2′-bipyridine), whose structures are shown in Chart 2. In all cases, the quantum yields of CO_2_ reduction were very high.

## Results and discussion

### Design and synthesis of the Re-rings

The design of the new Re-rings was based on our previous work, which clearly demonstrated the relationship between the ring structures (*i.e.*, the size of the ring and the type of the bridging bisphosphine ligand P–P) and their photophysical and electrochemical properties.^16–18^ The trinuclear Re-ring connected with a phenylene spacer in the bisphosphine ligand exhibited both a long lifetime and a strong oxidation power in the excited state. The corresponding OERS, which are important intermediates in the redox-photosensitized reactions, were relatively stable. In order to tune these properties in a more desirable fashion, further structural modification can be performed on the bpy ligand. Namely, we prepared a series of new trinuclear Re-rings bearing 2,2′-bipyridine ligands with electron-donating substituents and connected with a phenylene spacer, as shown in Chart 1.

**Scheme 1:**
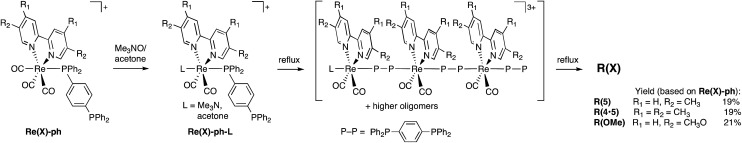
Synthetic route for the Re-rings.

We successfully developed a new one-pot synthetic method for these Re-rings without using photochemical reactions. In the previously reported synthesis (Scheme S1, ESI†), the photochemical reaction was an essential procedure for removing the edge CO ligands from the corresponding linear-shaped Re(i) multinuclear complexes. In the synthesis of the **R(4·5)** and **R(OMe)** rings, however, this reaction could not be used due to the fast photochemical decomposition of the linear-shaped Re(i) trimers. In the new synthetic method, the oligomerization of the mononuclear Re(i) complex and the cyclization process can be completed simultaneously. A tricarbonyl Re(i)–diimine complex was reacted with Me_3_NO, which is reported to be an effective decarbonylation reagent for metal carbonyl complexes,^19,20^ affording the biscarbonyl-Re(i) mononuclear complex as a building block with a labile ligand (**Re(X)-ph-L**, L = solvent molecule, Scheme 1). These reactions were instantaneous even under mild conditions and proceeded quantitatively. Prolonged reflux without any additional reagents led to both coupling and cyclization, affording the corresponding trinuclear Re-ring as the major product, with larger linear and eventually ring-shaped Re(i) multinuclear complexes as minor products (Fig. S1, ESI†). The Re-rings **R(4·5)**, **R(OMe)** and **R(5)** were successfully isolated from the reaction mixtures using size exclusion chromatography in *ca.* 20% yield, which is highly comparable with or better than the total yields of the multi-step synthetic strategy, including the photochemical reaction (Scheme S1, ESI†).

### Photophysical properties

**Fig. 1:**
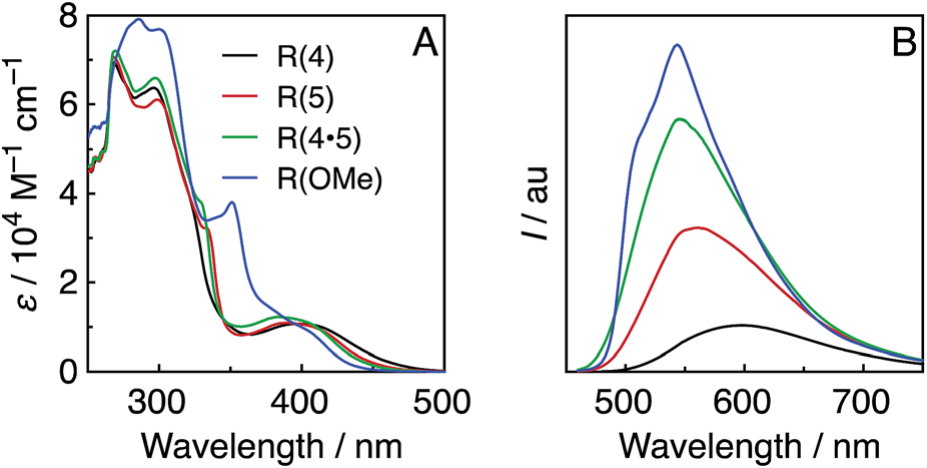
UV-vis (A) and emission (B) spectra of the Re-rings in DMF under Ar. The emission spectra were corrected to the absorbed photons at *λ*_ex_ = 400 nm.

Fig. 1A shows the UV-vis absorption spectra of the Re-rings, measured in DMF. In addition to an interligand transition (IL) of the bpy ligands at *λ*_abs_ ≈ 300 nm, a well-defined and intense ^1^MLCT absorption band centred at *λ*_abs_ ≈ 400 nm was observed for the Re-rings with methyl-substituted bpy ligands. In contrast, **R(OMe)** displayed both a strong absorption at *λ*_abs_ ≈ 300 to 350 nm and a shoulder peak at *λ*_abs_ ≈ 380 to 410 nm.

All of the Re-rings emitted strongly in DMF, even at room temperature (Fig. 1B). The emissive state is attributable to the ^3^MLCT excited state in the cases of the Re-rings with methyl-substituted bpy ligands because of their broad and non-vibrational shapes. However, the emission spectrum of **R(OMe)** showed a vibrational structure (*λ*_em_ ≈ 540 nm, 510 nm (sh)), which indicates that the emissive state contained not only ^3^MLCT but also ^3^IL character.

Table 1 summarizes the photophysical properties of the Re-rings. Both the emission quantum yields (*Φ*_em_) and the emission lifetimes (*τ*_em_) concomitantly increased in the case of the Re-ring with a higher emission energy. It is noteworthy that the emission quantum yield of **R(OMe)** is the highest among the reported Re(i) complexes (*Φ*_em_ = 0.66), and the lifetime of the excited **R(OMe)** was also very long (*τ*_em_ = 7.8 μs), mainly due to the contribution of the ^3^IL state. These absorption and emission properties were not affected by the presence of CO_2_.

**Table 1:** Photophysical properties of **R(X)** measured in DMF or DMA at 25 °C

	Solv.	*λ*_abs_ ^*a*^/nm (*ε*/10^3^ M^–1^ cm^–1^)	*λ*_em_ ^*b*^ /nm	*Φ*_em_ ^*b*^	*τ*_em_ ^*c*^ /μs
**R(4)**	DMF	398 (10.9)	598	0.12	1.57
**R(5)**	DMF	390 (11.5)	561	0.36	2.32
	DMA	389 (11.9)	557	0.31	2.04
**R(4·5)**	DMF	384 (11.9)	545	0.60	3.58
**R(OMe)**	DMF	380^br^ (12.0)	543	0.66	7.77
**R(5)-e** ^*d*^	DMF	409 (12.5)	571	0.41	5.40

^*a*^MLCT band.

^*b*^ *λ*_ex_ = 400 nm.

^*c*^ *λ*_ex_ = 401 nm.

^*d*^ **R(5)-e** = [{Re(5dmb)(CO)_2_(*η* ^2^-dppe)}_3_](PF_6_)_3_ (5dmb = 5,5′-dimethyl-2,2′-bipyridine, dppe = PPh_2_(CH_2_)_2_PPh_2_).^16^ ^br^ broad.

### Electrochemical properties

**Fig. 2:**
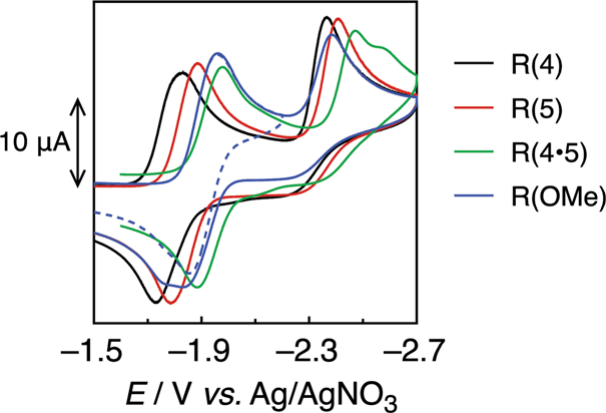
Cyclic voltammograms of **R(X)** (0.5 mM) in DMF under Ar, measured with a 100 mV s^–1^ sweep rate.

Fig. 2 shows the cyclic voltammograms of the Re-rings, where one reversible reduction wave was observed at *E*red1/2 = –1.82 to –1.91 V *vs.* Ag/AgNO_3_. This is attributed to a 3-electron reduction process,^21^ except for **R(4)**, where each electron was supplied to the diimine ligand in each Re(i) unit of the Re-ring (bpy/bpy˙^–^). This strongly suggests that the Re units in the Re-ring have no strong electronic interaction with each other.

On the contrary, the corresponding wave of **R(4)** could be deconvoluted with two Gaussian functions with a 2 : 1 intensity ratio (Fig. S2, ESI†). Hence, some electronic interplay between the units may occur in **R(4)**, just as we have also observed in smaller Re rings with shorter bridging ligands.^17,18^

The second irreversible waves at *E*_p_ = –2.4 to –2.5 V *vs.* Ag/AgNO_3_ correspond to the reduction of the metal centre. Table 2 summarizes the electrochemical properties.

**Table 2:** Electrochemical and quenching properties of **R(X)** measured in DMF or DMA at 25 °C

	Solv.	*E* red 1/2 ^*a*^/V (*n*e^–^)^*b*^	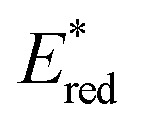 ^*c*^ /eV	*K*_SV_ ^*d*^ /M^–1^	*k*_q_ ^*e*^/10^6^ M^–1^ s^–1^	*η*_q_ ^*f*^
**R(4)**	DMF	–1.73 (2)	0.76^*g*^	4.5	2.9	0.85
		–1.80 (1)				
**R(5)**	DMF	–1.82	0.75	11.4	4.9	0.93
	DMA	–1.74	0.85	17.5	8.6	0.96
**R(4·5)**	DMF	–1.91	0.74	5.2	1.5	0.87
**R(OMe)**	DMF	–1.89	0.63	26.0	3.4	0.97
**R(5)-e** ^*h*^	DMF	–1.83 (2)	0.67^*g*^	3.5	0.7	0.81
		–1.91 (1)				

^*a*^First reduction potential *vs.* Ag/AgNO_3_, determined from the DPV peaks.

^*b*^ *n* = 3 except for **R(4)** and **R(5)-e**.

^*c*^ 
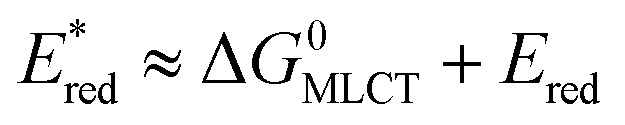
.

^*d*^Stern–Volmer constants obtained from quenching experiments of emission by TEOA.

^*e*^ *k*_q_ = *K*_SV_/*τ*_em_.

^*f*^[TEOA] = 1.256 M.

^*g*^Based on the first *E*_red_.

^*h*^From ref. 16.

### Reductive quenching and formation of one-electron-reduced species

The redox properties of the ^3^MLCT excited state, *i.e.*, the values of 
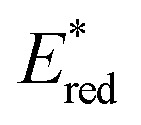
 were calculated on the basis of *E*red1/2 (Table 2) and Δ*G*0MLCT (these values were obtained using Franck–Condon analysis, see ESI† for a detailed description).

**Fig. 3:**
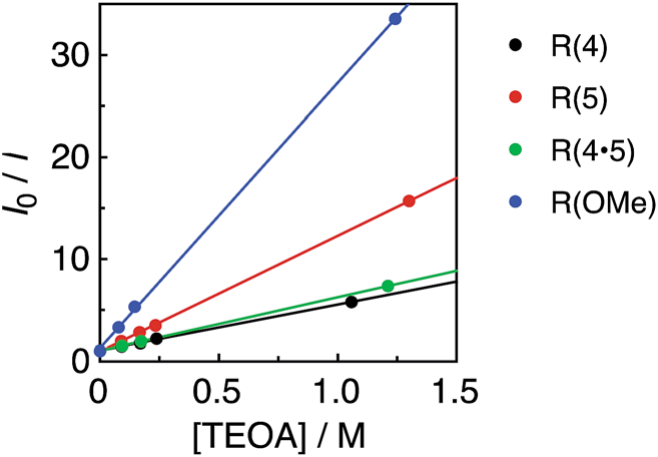
Stern–Volmer plots obtained from the emission quenching of **R(X)** using TEOA in DMF under Ar; *λ*_ex_ = 400 nm.

All of the Re-rings have sufficiently positive values of 
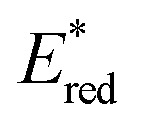
 to use triethanolamine (TEOA) as a reductant, which has often been used in various photocatalytic reactions. However, the quenching efficiencies of the excited states of some other PSs, such as Ru(ii)-trisdiimine complexes, are low because of the relatively weak reducing power of TEOA (*E*_ox_ = 0.51 V *vs.* Ag/AgNO_3_),^22^ or the too short a lifetime of the metal complex. Fig. 3 shows the linear Stern–Volmer plots of the emission intensity of the Re-rings in the presence of increasing concentrations of TEOA, which allowed us to obtain the Stern–Volmer constants, *K*_SV_, and the quenching rate constants, *k*_q_, listed in Table 2. All of the Re-rings with the phenylene chain exhibited higher quenching fractions (*η*_q_) in a DMF–TEOA (5 : 1 v/v) solution compared to the corresponding mononuclear Re(i)–bisphosphine complexes and even compared to other rings with a saturated alkyl spacer in P–P. For example, the *k*_q_ of **R(5)** was 7 times larger than that of the corresponding Re-ring with ethylene chains (**R(5)-e**) instead of phenylene chains.

**Fig. 4:**
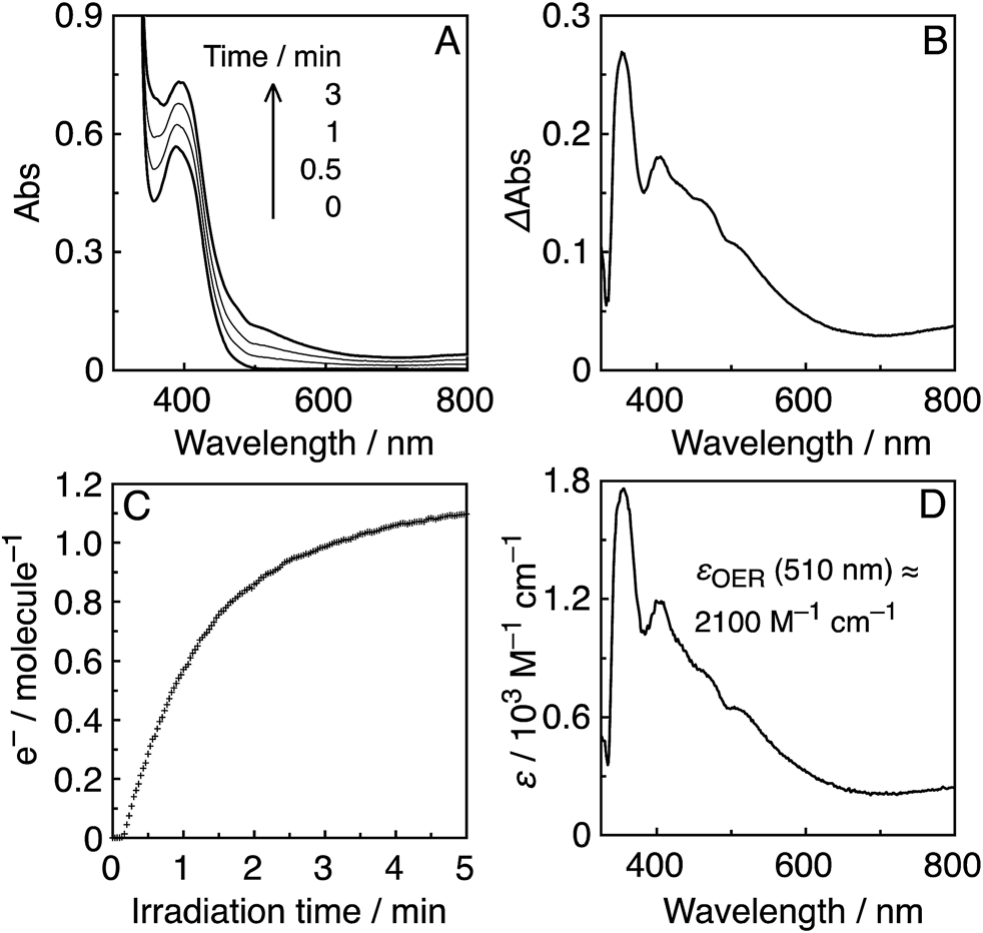
(A) UV-vis absorption spectral changes of an Ar-saturated DMF–TEOA (5 : 1 v/v) solution containing **R(5)** (0.05 mM) under irradiation at *λ*_ex_ = 436 nm (5.6 × 10^–9^ einstein per s). (B) Differential absorption spectrum before irradiation and after 3 min irradiation. (C) Time course of accumulated electrons in **R(5)**, compensated for the inner filter effect. (D) Differential absorption spectrum of **R(5)** before and after bulk electrolysis in an Ar-saturated DMF solution at –1.95 V *vs.* Ag/AgNO_3_.

Irradiation of an Ar-saturated DMF–TEOA (5 : 1 v/v) solution containing the Re-rings at *λ*_ex_ = 436 nm caused changes in the UV-vis absorption spectra. Fig. 4A shows the case of **R(5)**, where the change is attributed to the formation and accumulation of the OER species **R(5)**˙^–^ in the solution because the differential spectrum before and after the irradiation (Fig. 4B) was very similar to that of **R(5)**˙^–^ obtained using bulk electrolysis (Fig. 4D). The formation quantum yield of **R(5)**˙^–^ (*Φ*_OER_) was 1.2, and further irradiation induced an accumulation of about 1.1 electrons into one molecule of **R(5)** (Fig. 4C).

In the cases of the other Re-rings, the corresponding OERS were also accumulated in the solution using irradiation in the presence of TEOA (Fig. S3 and S4, ESI†). These results strongly indicate that the photochemical formation of the OERS of the Re-rings efficiently proceeds using TEOA as the reductant, and the OERS are relatively stable in anaerobic solution even under irradiation. It is noteworthy that the OERS also accumulated even under a CO_2_ atmosphere, with similar *Φ*_OER_ in the absence of the catalysts that were used for CO_2_ reduction, as described below.

### Photocatalytic reactions

Various metal complex catalysts for CO_2_ reduction, *e.g.*, Ru(ii),^23–26^ Co(ii),^27^ Ni(ii),^28,29^ Re(i),^30–33^ Fe(ii),^34,35^ Os(ii),^36^ Ir(iii)^37^ and Mn(i),^38^ have been reported mostly with Ru(ii)–trisdiimine complexes, typically [Ru(bpy)_3_]^2+^ or [Ru(4dmb)_3_]^2+^ (4dmb = 4,4′-dimethyl-2,2′-bipyridine) as PSs. We selected three efficient and widely studied catalysts, *i.e.*, Re(i)-, Ru(ii)- and Mn(i)-diimine carbonyl complexes, to investigate photocatalytic CO_2_ reduction using the Re-rings as PSs.

#### Photocatalytic reaction with *fac*-[Re(bpy)(CO)_3_(CH_3_CN)]^+^

Because the excited states of the Re-rings with the phenylene chains have stronger oxidation powers compared to the previously reported ring with ethylene chains, which requires the usage of 1,3-dimethyl-2-phenyl-2,3-dihydro-1*H*-benzo[*d*]imidazole (BIH) as an electron donor,^39^ we could employ triethanolamine (TEOA), which is commonly used in various photocatalytic reactions but is a weaker reductant than BIH. *fac*-[Re(bpy)(CO)_3_(CH_3_CN)]^+^ (**Re-ACN**) was used as a catalyst; it is quantitatively converted into the CO_2_ adduct (**Re-OCO(O)NR_2_**) with the aid of TEOA (eqn (1)) under the photocatalytic reaction conditions.^40^ Although **Re-ACN** is not the real catalyst, we use this nomenclature in the following text because it was used as the starting complex.1
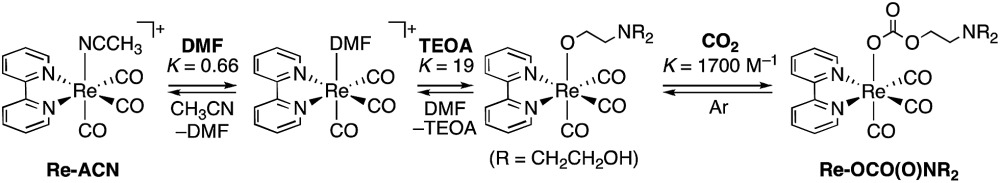



In a typical run (eqn (2)), a CO_2_ saturated DMF–TEOA (5 : 1 v/v) mixed solution containing both **R(5)** and **Re-ACN** in an equimolar ratio (0.05 mM) was irradiated at *λ*_ex_ = 436 nm (5.7 × 10^–9^ einstein per s), where 87% of the irradiated photons were absorbed by **R(5)**, giving CO selectively.2
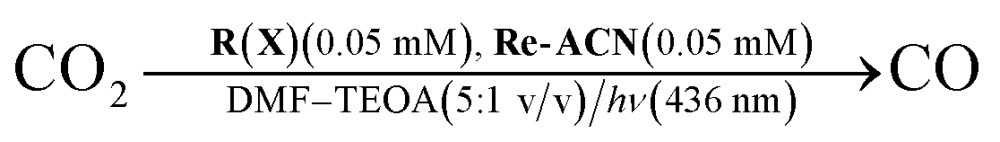



The quantum yield (*Φ*_CO_, eqn (3)) was 0.61 and the TON (eqn (4)) of CO formation (TON_CO_) was 71 after 15 h of irradiation (entry 2, Table 3).

3 *Φ*_product_ = product [mol]/total absorbed photons by the solution [einstein]

4 TON_product_ = product [mol]/catalyst [mol]

**Table 3:** Photocatalytic formation of CO at 1 h or 15 h of irradiation and quantum yields of CO formation under irradiation at *λ*_ex_ = 436 nm or 405 nm^*a*^

Entry	PS	*n*_CO_/μmol (TON_CO_)^*b*^		*Φ*_CO_ ^*c*^	
		1 h	15^*d*^ h	*λ*_ex_ ^*b*^ = 436 nm	*λ*_ex_ ^*e*^ = 405 nm
1	**R(4)**	5.42 (27)	19.68 (98)	0.53	—^*f*^
2	**R(5)**	4.44 (22)	14.2 (71)	0.61	0.67
3^*g*^	**R(5)**	1.28 (6)	1.52 (8)	—^*f*^	
4	**R(4·5)**	3.95 (20)	6.31 (32)	0.60	0.61
5	**R(OMe)**	2.13 (11)	9.63 (48)	0.60	0.74
6	**R(5)-e**	6.37 (32)	6.72 (34)	0.53	—^*f*^
7	None	0 (0)	0.32 (1.6)	—^*f*^	

^*a*^Photocatalytic CO_2_ reduction using a DMF–TEOA mixture (5 : 1 v/v) containing **R(X)** (0.05 mM) as a PS and **Re-ACN** as the catalyst.

^*b*^Under *λ*_ex_ = 436 nm with 5.7 × 10^–9^ einstein per s light intensity, [**Re-ACN**] = 0.05 mM.

^*c*^± 2%.

^*d*^Level off.

^*e*^1.3 × 10^–9^ einstein per s light intensity, [**Re-ACN**] = 0.025 mM.

^*f*^Not determined.

^*g*^Without **Re-ACN**.

The use of other PSs, **R(4·5)** and **R(OMe)**, instead of **R(5)** gave similar *Φ*_CO_ values; however, TON_CO_ decreased (entries 4 and 5, Table 3). In the case of using **R(4)**, *Φ*_CO_ was lower, while TON_CO_ was slightly higher than the others (entry 1, Table 3). Note that the photocatalyses of the systems using these new Re-rings as the PS were higher than that using **R(5)-e** (entry 6, Table 3), which was the most efficient PS for CO_2_ reduction using BIH, which is a much stronger reductant than TEOA, in the reported system. The lower quenching efficiency of the ^3^MLCT excited state of **R(5)-e** by TEOA – in other words, its weaker oxidation power – should be a main reason for this lower *Φ*_CO_.

Irradiation at a slightly shorter wavelength with a lower light intensity (*λ*_ex_ = 405 nm, instead of 436 nm), and a half-molar ratio of the catalyst (0.025 mM) increased the *Φ*_CO_ up to 0.74 (Table 3), mainly because of the reduced inner-filter effect of the catalyst, which affects the number of photons absorbed by the photocatalytic mixture (Table S4, ESI†). In addition, lower light intensity may sometimes have an influence on the quantum yield. This phenomenon was observed in the cases using 436 nm-irradiation light, for example, the **R(5)**/**Re-ACN** photocatalytic system gave slightly higher *Φ*_CO_ (0.68), under irradiation with a 3.5-fold lower light intensity (1.65 × 10^–9^ einstein per s).

**Fig. 5:**
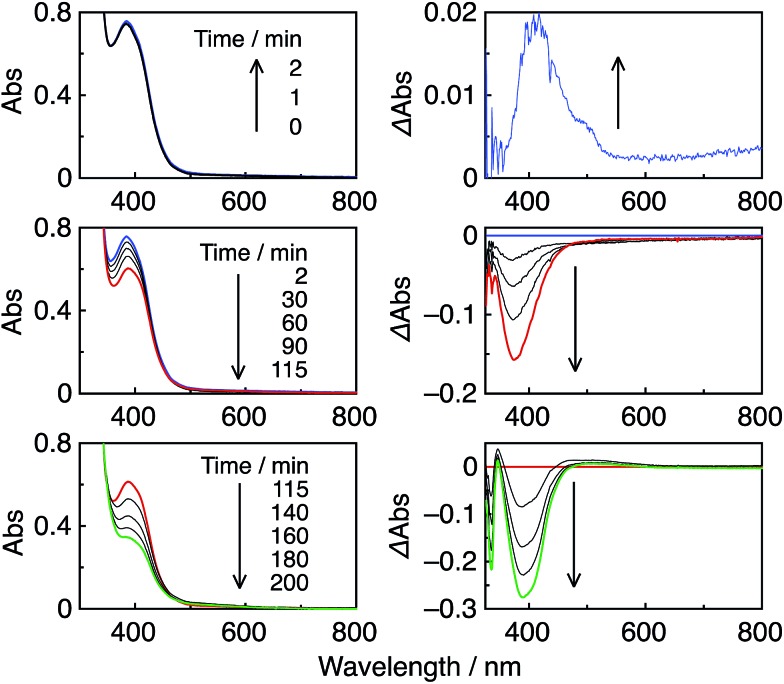
UV-vis (left) and the corresponding differential absorption spectral changes (right) during the irradiation of a CO_2_-saturated DMF–TEOA (5 : 1 v/v) solution containing **R(5)** (0.05 mM) as a PS and **Re-ACN** (0.05 mM) as the catalyst, under irradiation at *λ*_ex_ = 436 nm (5.7 × 10^–9^ einstein per s) in the initial stage (top) and over 3.5 h irradiation (middle and bottom).

These high values of *Φ*_CO_ can be attributed to several factors. One important reason is likely the high formation yields of the OERS of the Re-rings *via* reductive quenching of the excited Re-rings with TEOA because of their long lifetimes and strong oxidation powers in the excited state, as described in the previous section. Another reason is the rapid electron transfer process from **R(X)**˙^–^ to the catalyst, which suppresses the accumulation of the OERS of the Re-rings. Since accumulation of the OERS induces the inner-filter effect owing to their strong absorption in the visible region (Fig. 4), it should decrease the number of photons absorbed by the (not-reduced) Re-rings in the photocatalytic mixture. This makes the apparent quantum yield lower. Fig. 5 shows the UV-vis absorption spectral changes of the photocatalytic reaction solution containing the **R(5)** PS with the **Re-ACN** catalyst, where accumulation of the OERS of **R(5)** was not observed. Note that most of the **R(5)** was converted to the corresponding OERS within 3 min of irradiation in the absence of the catalyst (Fig. 4). These results clearly indicate that electron transfer from the OERS of **R(5)** to the catalyst was not the rate-limiting step. Since the reduction potential of **R(5)** (Table 2) was more negative than the onset potential of **Re-OCO(O)NR_2_** (more positive than –1.8 V *vs.* Ag/AgNO_3_ in DMF–TEOA, Fig. S5, ESI†), the electron scavenging process from the OERS of **R(5)** using the catalyst has an exergonic character.

Similar results were obtained in the photocatalytic reactions using the other PSs except **R(4)** (Fig. S6 to S8, ESI†). A very small amount of the OERS of **R(4)** accumulated within 2 min of irradiation, which suggests a slower electron transfer process for the catalyst compared to the other Re-rings, probably because of the more positive redox potential of **R(4)**. This may be a reason why *Φ*_CO_ decreased in the case using **R(4)** compared to the other Re-rings; another reason is likely the lower quenching ratio of the excited state of **R(4)** using TEOA (Table 2).

**Fig. 6:**
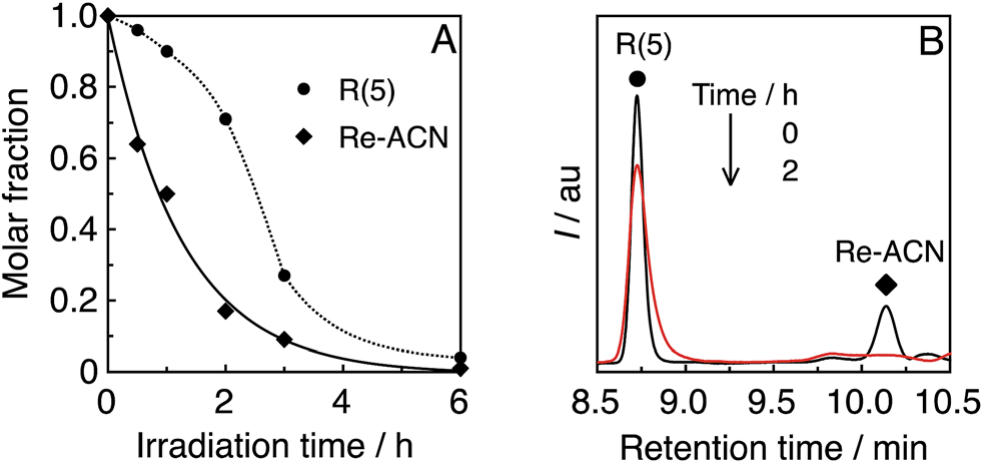
(A) Residual **R(5)** and **Re-ACN** in solution during the photocatalytic reaction, obtained using UPLC analysis (Fig. S9, ESI†). (B) UPLC chart of the photocatalytic system **R(5)**/**Re-ACN** before and after 2 h of irradiation; *λ*_det_ = 350 nm.

Until reaching 1 h of irradiation, **R(5)** functioned stably as the PS; however, a longer irradiation time induced the decomposition of **R(5)** after most of the catalyst was already decomposed (see the result after 2 h of irradiation as an example in Fig. 6B). Therefore, the Re-rings should be durable PSs in the presence of the catalyst.

In the absence of the Re-ring, only a trace amount of CO was observed (entry 7, Table 3); hence the catalyst without the Re-ring could not photocatalyze CO_2_ reduction under these reaction conditions. In the case using solely **R(5)** without the catalyst, a certain amount of CO was produced (entry 3, Table 3: TON_CO_ = 6 for 1 h irradiation, and 8 even for 15 h irradiation). Note that the Re-ring has 6 CO ligands, the decomposition of **R(5)** should yield some CO molecules. Although the decomposition products of **R(5)** might work as catalysts for CO_2_ reduction with the residual **R(5)** as a PS, their contribution to the photocatalytic reaction should be very low because the CO formation mostly stopped after 1 h of irradiation.

**Fig. 7:**
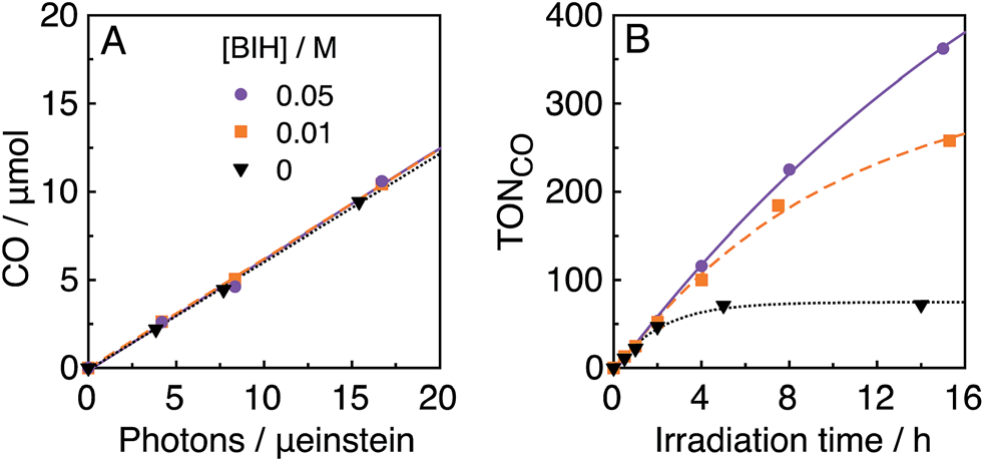
Absorbed photon-dependence (A) and time courses (B) of photocatalytic CO evolution using **R(5)** (0.05 mM) as the PS and **Re-ACN** (0.05 mM) as the catalyst with various concentrations of BIH in DMF–TEOA (5 : 1 v/v) under 436 nm light irradiation of 5.7 × 10^–9^ einstein per s intensity.

In the photocatalytic system of **R(5)**/**Re-ACN**, usage of BIH instead of TEOA as the reductant did not affect the efficiency of the photocatalytic reaction, *i.e.*, *Φ*_CO_ did not change in the presence of various concentrations of BIH (*Φ*_CO_ ∼ 0.6, Fig. 7A). However, TON_CO_ drastically increased with higher concentrations of BIH, as illustrated in Fig. 7B. The total amount of produced CO was similar or even greater than the initial amount of BIH after a long irradiation, *e.g.*, the amount of evolved CO was 50 μmol after 15 h of irradiation in the presence of 40 μmol of BIH, where both BIH and TEOA should work as reductants. It has been reported that BIH is a quantitative 2e^–^ donor (eqn (5)), and its oxidation product (BI^+^) did not impede the photocatalytic reduction of CO_2_.^39^ Therefore, accumulation of the oxidation products of TEOA in the reaction solution may disturb the photocatalytic reaction in some process(es) because CO formation levelled off after TON_CO_ reached 70 in the presence of only TEOA as the reductant.5
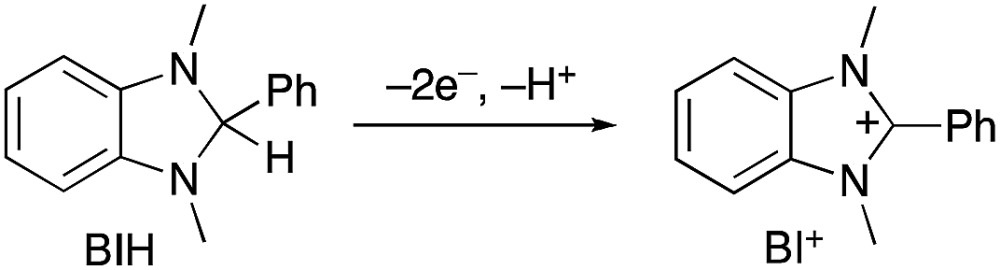



#### Photocatalytic reactions with *trans*(Cl)–Ru(dtbb)(CO)_2_Cl_2_

Recently, *trans*-Ru(N^N)(CO)_2_Cl_2_ (N^N = diimine ligand) has been often used as a catalyst for CO_2_ reduction, giving CO under neutral and acidic conditions or HCOOH under basic conditions.^25,41,42^ Since this type of complex usually suffers undesirable reductive polymerization during the photocatalytic reaction, which slows or disables the reaction progress, we chose an Ru(ii) catalyst with bulkier *tert*-butyl groups on the diimine ligand, *trans*(Cl)–Ru(dtbb)(CO)_2_Cl_2_ (**Ru(*t*Bu)-Cl_2_**, Chart 2). Its absorption and electrochemical properties in DMA–TEOA are shown in the ESI (Fig. S10 and S14B†).

**Fig. 8:**
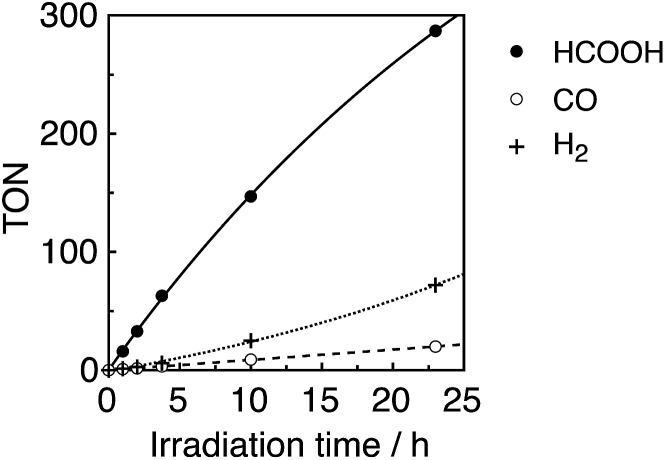
Photocatalytic CO_2_ reduction using **R(5)** (0.05 mM) as the PS and **Ru(*t*Bu)-Cl_2_** (0.05 mM) as the catalyst in DMA–TEOA (5 : 1 v/v) under 436 nm light irradiation of 4.2 × 10^–9^ einstein per s intensity.

A CO_2_-saturated DMA–TEOA (5 : 1 v/v) mixed solution containing an equimolar ratio (0.05 mM) of **Ru(*t*Bu)-Cl_2_** catalyst and **R(5)** PS was irradiated under 436 nm light (4.2 × 10^–9^ einstein per s), where 98% of the photons were absorbed by **R(5)**. Formic acid was evolved as a major product, accompanied by H_2_ and CO as minor products (Fig. 8). The turnover number (TON) of HCOOH reached 290, and the TONs for H_2_ and CO were around 70 and 20, respectively, after 23 h irradiation (Table 4).

**Table 4:** Photocatalytic CO_2_ reduction using **R(5)** as the PS with **Ru(*t*Bu)-Cl_2_** or **Mn(*t*Bu)-ACN** as the catalyst^*a*^

	BI(OH)H	TON			*Φ* ^*b*^
	M	HCOOH	CO	H_2_	HCOOH
**Ru(*t*Bu)-Cl_2_** ^*c*^	0	290	20	72	0.58
	0.03	280	16	49	0.47^*e*^
**Mn(*t*Bu)-ACN** ^*d*^	0	85	32	Traces	0.48
	0.03	60	80	Traces	0.37^*e*^

^*a*^Photocatalytic CO_2_ reduction using DMA–TEOA mixed solution (5 : 1 v/v) containing **R(5)** (0.05 mM) as the PS and the catalyst (0.05 mM) under 436 nm light irradiation.

^*b*^±2%.

^*c*^TON at 23 h of irradiation with 4.2 × 10^–9^ einstein per s light intensity.

^*d*^TON at 12 h of irradiation with 5.3 × 10^–9^ einstein per s light intensity.

^*e*^Not taking into account absorption of BI(O^–^)^+^.

**Fig. 9:**
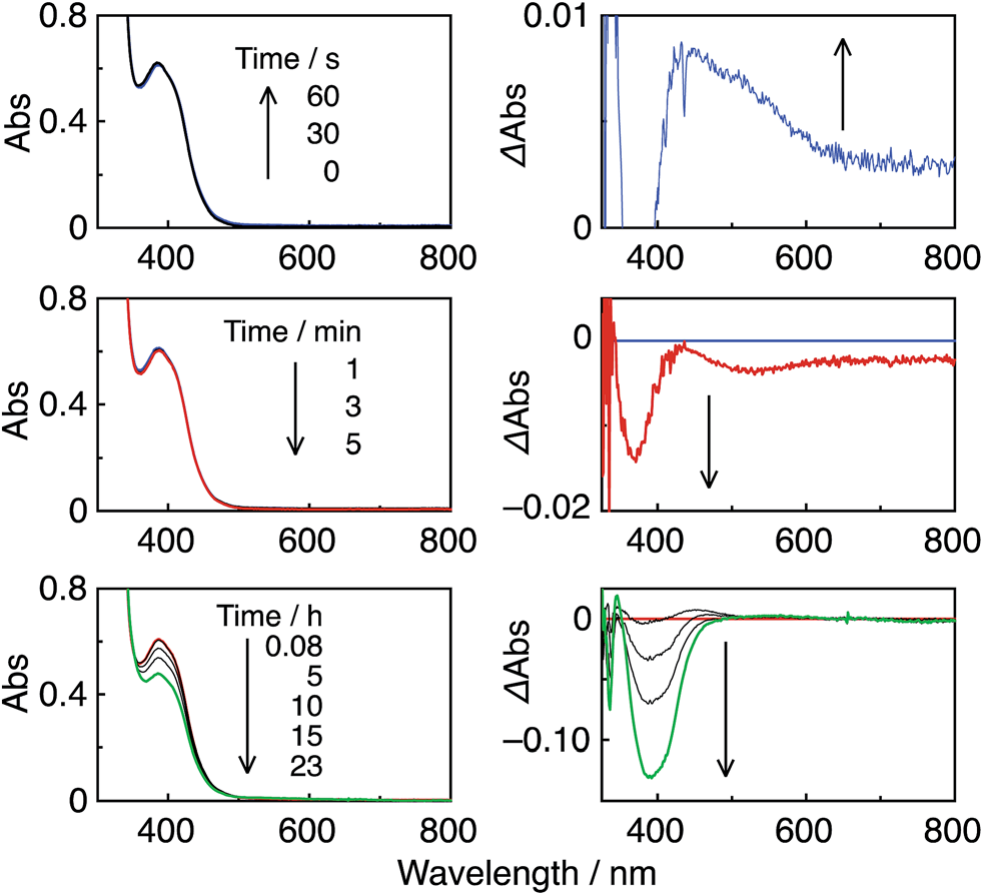
UV-vis (left) and the corresponding differential absorption spectral changes (right) during the irradiation of a CO_2_-saturated DMA–TEOA (5 : 1 v/v) solution containing **R(5)** (0.05 mM) as the PS and **Ru(*t*Bu)-Cl_2_** (0.05 mM) as the catalyst, under irradiation at *λ*_ex_ = 436 nm (4.2 × 10^–9^ einstein per s).

In the photocatalytic reaction, **R(5)** was very durable, and *ca.* 80% of **R(5)** remained even after 23 h of irradiation (Fig. 9, bottom). The quantum yield of HCOOH (*Φ*_HCOOH_) formation was 58% and was independent of the light intensity. This quantum yield is the highest value reported to date for photocatalytic systems for the reduction of CO_2_ to HCOOH.

It should be noted that most of the reported photocatalytic systems comprising mononuclear PSs and a catalyst usually contain a much larger amount of PS than the catalyst to supply electrons to the catalyst more rapidly and/or to prevent light absorption by the catalyst. Although the same concentration of the PS **R(5)** as the catalyst (**Ru(*t*Bu)-Cl_2_**) was used in this photocatalytic reaction, only a small amount of dimer [Ru^I^(dtbb)(CO)_2_L]_2_, possibly including OER of the Ru(ii) catalyst, was observed at a very early stage (Fig. 9, top). Further irradiation caused a decrease in dimer concentration (Fig. 9, middle and bottom), and the polymer of the Ru complex was not detected during the photocatalytic reaction. Hence, Ru-polymerization was efficiently suppressed. However, steric hindrance of the *tert*-butyl groups was not the main factor for the inhibition of polymerization because a similar experiment under Ar atmosphere instead of CO_2_ induced quantitative formation of the dimer of the Ru complex at a very early stage (Fig. S11, ESI†), followed by its polymerization. Conversely, during the photocatalytic reaction under a CO_2_ atmosphere, neither the OERS of the Re-ring (**R(5)**˙^–^) nor the Ru polymer were detected, suggesting that the reaction of **R(5)**˙^–^ with the catalyst (and/or the dimer) and the subsequent reaction with CO_2_ were very efficient and fast.

No change in TON_HCOOH_ was observed upon addition of a stronger electron donor, *i.e.*, 1,3-dimethyl-2-(*o*-hydroxyphenyl)-2,3-dihydro-1*H*-benzo[*d*]imidazole (BI(OH)H).^43^ Moreover, its oxidized product, BI(O^–^)^+^ (eqn (6)), caused a strong inner filter effect due to its absorption at *λ*_ex_ 436 nm (Fig. S12, ESI†), thereby lowering the apparent *Φ*_HCOOH_ (Table 4).

Employing another Re-ring with a more negative reduction potential (**R(4·5)**) and **Ru(*t*Bu)-Cl_2_** catalyst did not improve the photocatalytic performance (Fig. S13 and Table S5, ESI†).6
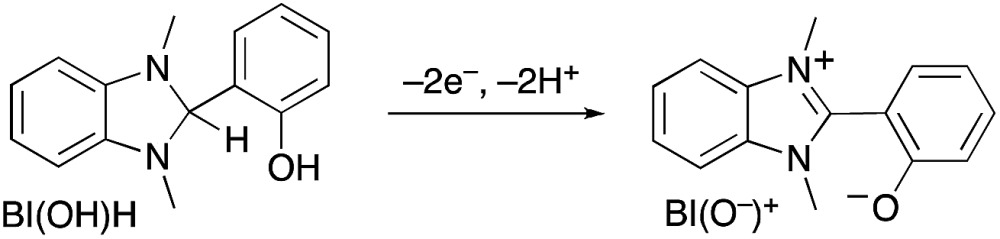



#### Photocatalytic reaction with *fac*-[Mn(dtbb)(CO)_3_(CH_3_CN)]^+^

As an earth-abundant metal catalyst, *fac*-Mn(N^N)(CO)_3_Br has attracted attention mainly in electrochemical systems for the reduction of CO_2_ to CO.^44,45^ Recently, the Mn complex was also applied to photocatalytic CO_2_ reduction with a [Ru(N^N)_3_]^2+^ PS, where the main product was HCOOH.^38,46^ However, the efficiencies of the photocatalytic systems are not very high (*Φ*_HCOOH_ = 0.05 to 0.14).

We chose a Mn(i) complex with a dtbb ligand, as for the Ru(ii) catalyst in the previous section, with a CH_3_CN axial ligand, *fac*-[Mn(dtbb)(CO)_3_(CH_3_CN)]^+^ (**Mn(*t*Bu)-ACN**, Chart 2). The UV-vis absorption spectrum of **Mn(*t*Bu)-ACN** in DMA–TEOA under CO_2_ is shown in Fig. S14C (ESI†) and the first reduction potential was *E*redp = –1.68 V *vs.* Ag/AgNO_3_ under a CO_2_ atmosphere (Fig. S15, ESI†).

**Fig. 10:**
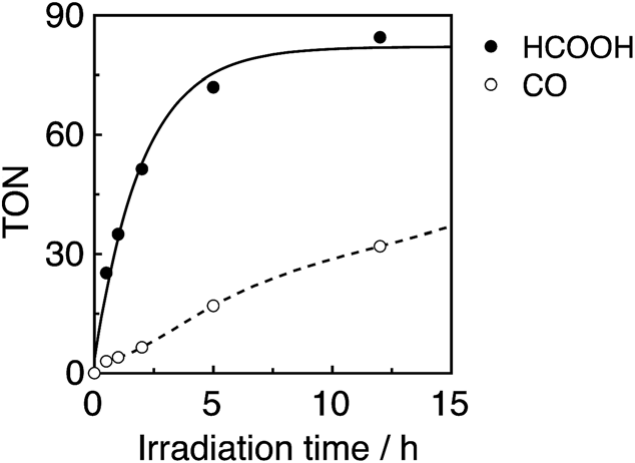
Photocatalytic CO_2_ reduction using **R(5)** (0.05 mM) as the PS and **Mn(*t*Bu)-ACN** (0.05 mM) as the catalyst in DMA–TEOA (5 : 1 v/v) under 436 nm light irradiation of 5.3 × 10^–9^ einstein per s intensity.

In a typical run, a CO_2_-saturated DMA–TEOA (5 : 1 v/v) mixed solution containing both **R(5)** as the PS and **Mn(*t*Bu)-ACN** as a catalyst in an equimolar ratio (0.05 mM) was irradiated at *λ*_ex_ = 436 nm (5.3 × 10^–9^ einstein per s), of which 72% was absorbed by **R(5)** in at least the starting stage of the photocatalytic reaction. Formic acid was evolved as the major product, with an overall TON of 85, and accompanied by CO (Fig. 10).

**Fig. 11:**
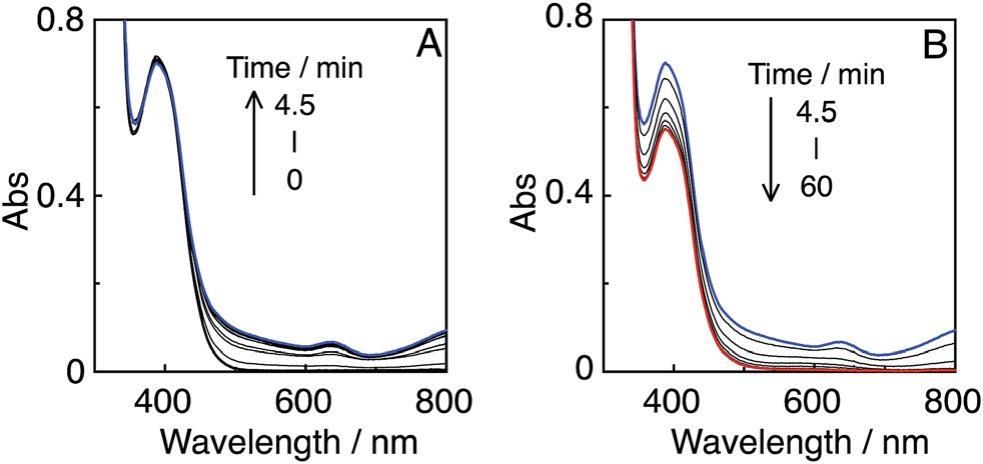
UV-vis absorption spectral changes of a CO_2_-saturated DMA–TEOA (5 : 1 v/v) solution containing **R(5)** (0.05 mM) as the PS and **Mn(*t*Bu)-ACN** (0.05 mM) as the catalyst, under irradiation at *λ*_ex_ = 436 nm (5.3 × 10^–9^ einstein per s) in the initial stage (A) and over 1 h irradiation (B).

In the initial stage, the UV-vis absorption spectral changes accurately indicated dimerization of the Mn complex, giving [Mn(dtbb)(CO)_3_]_2_ with a strong absorption in a wide range of the visible region (Fig. 11A). During the dimer formation, which increased up to 4.5 min of irradiation, the OERS of **R(5)** was not detected. These results clearly suggest that electron transfer from **R(5)**˙^–^ to the Mn complex proceeded rapidly, followed by dimerization of the OERS of the Mn complex. The absorption of the Mn dimer disappeared within 1 h of irradiation (Fig. 11B). The CO formation overlapped with the profile of the dimer lifetime, *i.e.*, the CO formation temporarily reached a plateau after the Mn-dimer decomposed (TON_CO_ = 4 at 1 h). Therefore, the Mn-dimer may function as a catalyst for CO formation. The formation of CO restarted after further irradiation. The time profile of the HCOOH formation was different from that of the CO formation (Fig. 10). The quantum yield of the HCOOH formation was 48% despite the inner filter effect of the dimer absorption. This is the highest value among reported photocatalytic systems using a Mn complex as the catalyst so far.

The photocatalytic reaction in the absence of the photosensitizer produced only 0.5 μmol of CO, and no HCOOH was observed, while the known photochemical *fac*- to *mer*- isomerisation with negligible Mn-dimer formation^47^ and simultaneous decomposition of the Mn(i) complex occurred rapidly (Fig. S16, ESI†).

The durability of the photocatalytic system was not improved by the addition of the stronger electron donor BI(OH)H. The formation of the Mn dimer and its persistence in solution were very similar to those in the system without BI(OH)H. Since the oxidized product, BI(O^–^)^+^, also caused an inner filter effect (Fig. S17, ESI†), the apparent quantum yield was lower when using BI(OH)H than when not using it.

Employing **R(4·5)**, which has a more negative reduction potential, did not improve the photocatalytic performance. The TONs for HCOOH and CO decreased compared to **R(5)**, owing to the lower stability of this ring (Fig. S18 and Table S5, ESI†).

## Experimental section

### Materials and methods

Dimethylformamide (DMF) and dimethylacetamide (DMA) were dried over 4 Å molecular sieves, distilled under reduced pressure and stored under Ar before use for a maximum of 1 week. Triethanolamine (TEOA) was distilled under reduced pressure (<1 Torr) and maintained under Ar before use. Other anhydrous solvents were purchased from commercial sources. All reactions were carried out under an inert atmosphere and dry conditions unless noted. Column chromatography was performed with Silica Gel 60 (40–50 μm, Kanto Chemical Co.). 2,2′-Bipyridine (bpy), 4,4′-dimetyl-2,2′-bipyridine (4dmb), 5,5′-dimetyl-2,2′-bipyridine (5dmb), 4,4′-di-*tert*-butyl-2,2′-bipyridine (dtbb) and other commercially available reagents were purchased from Kanto Chemical Co., Tokyo Kasei Co., Wako Pure Chemical Industries and Aldrich Chemical Co. and were used as received. Syntheses of *p*-bis(diphenylphosphino)benzene^48^ (ph), 5,5′-dimethoxy-2,2′-bipyridine^49^ (dmxb), 4,4′,5,5′-tetrametyl-2,2′-bipyridine^50^ (tmb), 1,3-dimethyl-2-phenyl-2,3-dihydro-1*H*-benzo[*d*]imidazole^51^ (BIH) and 1,3-dimethyl-2-(*o*-hydroxyphenyl)-2,3-dihydro-1*H*-benzo[*d*]imidazole^51^ (BI(OH)H) were reported elsewhere. The mononuclear acetonitrile-complex catalyst *fac*-[Mn(dtbb)(CO)_3_(CH_3_CN)]^+^ was prepared under dark conditions by following the procedure for *fac*-[Re(bpy)(CO)_3_(CH_3_CN)]^+^.^52^ *trans*(Cl)-[Ru(dtbb)(CO)_2_Cl_2_] was prepared according to the literature.^53^ All *fac*-Re(N^N)(CO)_3_Br-type^54^ and multinuclear Re(i)-type complexes^18,55^ were synthesized according to the literature. All target complexes were obtained as PF_6_^–^ salts.

Photochemical reactions were performed with a 500 W high-pressure mercury lamp EHBWI (Eikosha) with a uranium glass filter (>330 nm) in a Pyrex doughnut-form cell, with bubbling of N_2_ gas. During irradiation, both the reaction vessel and the light source were cooled with tap water. Separation of the larger complexes was achieved using size exclusion chromatography (SEC) using a pair of Shodex PROTEIN KW-2002.5 columns (300 mm × 20.0 mm i.d.) with a KW-LG guard-column (50 mm × 8.0 mm i.d.) and a JAI LC-9201 recycling preparative HPLC apparatus (Japan Analytical Industry Co.) with a JASCO 870-UV detector (the detection wavelength was chosen as 360 nm). The eluent was MeOH–CH_3_CN (1 : 1 v/v) containing CH_3_COONH_4_ (0.15 M) and the flow rate was 6.0 mL min^–1^.^56^ For analytical SEC, we used two sequentially connected Shodex KW-402.5-4F columns (300 mm × 4.6 mm i.d.), a KW400G-4A guard-column (10 mm × 4.6 mm i.d.), a JASCO 880-51 degasser, a 880-PU pump, a MD-2010 Plus UV-vis photodiode-array detector (*λ*_det_ = 360 nm) and a Rheodyne 7125 injector. The column temperature was maintained at 40 °C using a JASCO 860-CO column-oven. The eluent was MeOH–CH_3_CN (1 : 1 v/v) containing CH_3_COONH_4_ (0.5 M) and the flow rate was 0.2 mL min^–1^. For LC analysis of the photocatalytic reactions, we used a SHIMAZU UPLC Nexera X2 apparatus with a Waters Acquity SEC column (150 mm × 4.6 mm i.d.), a Shimadzu DGS-20A degasser, a LC-30AD pump, a SPD-M30A UV-vis photodiode-array detector and a Rheodyne 7125 injector. The column temperature was maintained at 40 °C using a JASCO 860-CO column-oven. The eluent was MeOH–CH_3_CN (1 : 1 v/v) containing CH_3_COONH_4_ (0.5 M), and the flow rate was 0.2 mL min^–1^. ^1^H-NMR and ^31^P-NMR spectra were acquired with a JEOL AL400, JEOL ECX400 or JEOL ECA400II spectrometer. Chemical shifts (*δ*/ppm) were referenced to the residual ^1^H-signals of the deuterated solvent (1.94 ppm for CD_3_CN and 2.05 ppm for CD_3_COCD_3_) and the ^31^P-signal of PF_6_^–^ (–145 ppm), respectively. All NMR spectra were recorded at room temperature. Electrospray ionization mass spectrometry (ESI-MS) was conducted on a Shimadzu LCMS-2010A mass spectrometer. Electrospray ionization time-of-flight mass spectrometry (ESI-TOFMS) was undertaken with a Waters LCT Premier instrument. FT-IR spectra were recorded with a JASCO FT/IR-610 spectrometer at 1 cm^–1^ resolution with a TGS detector. Electrochemical voltammetric techniques were performed using a CHI720D electrochemical analyzer (CH Instruments, Inc.) with a glassy-carbon working electrode (3 mm i.d.), an Ag/AgNO_3_ (10 mM) reference electrode and a Pt counter electrode. The supporting electrolyte was Et_4_NBF_4_ (0.1 M), which was dried under vacuum at 100 °C for 1 day prior to use. Bulk electrolysis was performed in a quartz UV-vis OTTLE cell (1.0 mm optical path length) equipped with a Pt-mesh working electrode, an Ag/AgNO_3_ (10 mM) reference electrode, and a Pt counter electrode. *In situ* UV-vis spectral changes were measured using a Photal MCPD-9800 spectrometer. The applied potential was controlled with a CHI720D electrochemical analyzer (CH Instruments, Inc.). The supporting electrolyte was Et_4_NBF_4_ (0.1 M), which was dried in vacuum at 100 °C for 1 day prior to use.

UV-vis absorption spectra were recorded with a JASCO V-565 or V-670 spectrophotometer. Emission spectra were recorded at 25 °C using a JASCO FP-6500 spectrofluorometer and were corrected for PMT response. Emission quantum yields were determined with a calibrated integrating sphere (Absolute PL Quantum Yield Measurement System C9920-01, Hamamatsu Photonics k.k.), comprising a Xe lamp as an excitation source and a multichannel spectrometer (C10027).^57^ Emission lifetimes were obtained at 25 °C using a HORIBA FluoroCube time-correlated single photon counting system. The excitation light source was a NanoLED-405L (401 nm, <200 ps). All measurements were performed in a quartz cubic cell (1 cm optical path length); the absorbances were adjusted to ≈0.1 at *λ*_ex_, and the solutions were degassed with Ar prior to the measurements.

Photochemical OERS formation experiments were performed in Ar-saturated DMF–TEOA (5 : 1 v/v) solution (4 mL) containing **R(X)** in a quartz cubic cell (1 cm optical path length). Photocatalytic reactions were performed in a CO_2_-saturated DMF–TEOA or DMA–TEOA (5 : 1 v/v) mixture containing **R(X)** and a catalyst in a quartz cubic cell (1 cm optical path length; 11 mL volume). In the case of the Re(i) or Mn(i) catalysts, a DMF or DMA solution, respectively, was prepared one night prior, starting from the corresponding acetonitrile complex. The sample solutions were irradiated using a high-pressure Hg lamp (Ushio USH-500SC) combined with a CuSO_4_·5H_2_O aqueous solution filter (250 g L^–1^, 5 cm pass length), a 436 or 405 nm band-pass filter (fwhm = 10 nm, Asahi Spectra Co.) and neutral density glass filters (Chuo Precision Industrial Co.) were used to adjust to the desired light intensity. *In situ* measurements of the UV-vis absorption spectra were conducted using a Photal MCPD-9800 or Photal MCPD-2000 spectrometer. The temperature of the reaction solution during the irradiation was maintained at 25 °C using an IWAKI CTS-134A constant-temperature system. The incident light intensity at 436 or 405 nm was determined using a K_3_[Fe(C_2_O_4_)_3_] chemical actinometer.^58^ The gaseous reaction products (CO and H_2_) were analysed using GC-TCD (GL Science GC323) with an active carbon column. HCOOH was analysed using a capillary electrophoresis system (CAPI-3300I, Otsuka Electronics Co.). As pretreatment for HCOOH quantification, the photocatalytic reaction solution was diluted by 10 times with H_2_O.

### Syntheses

#### General procedure for the synthesis of **Re(X)-ph**

A CH_2_Cl_2_ solution of *fac*-Re(N^N)(CO)_3_Br and AgOTf (1.05 eq.) was refluxed until the complete substitution of the Br^–^ ligand by OTf^–^ (3 to 7 h) as verified using TLC. The solution was filtered through a pad of Celite and evaporated to dryness. The crude *fac*-Re(N^N)(CO)_3_OTf and *p*-bis(diphenylphosphino)benzene (5 eq.) were refluxed in THF under an inert atmosphere in dim light for 2 d. The solvent was removed under reduced pressure; the crude solid was purified using column chromatography (SiO_2_, CH_3_CN–CH_2_Cl_2_ 1 : 5). **Re(X)-ph** was converted into PF_6_^–^ salt for the NMR, FT-IR and ESI-MS characterisation as follows. The solid was dissolved in a small amount of MeOH; then, a few drops of a saturated aqueous solution of NH_4_PF_6_ were added, leading to precipitation of the product. The solid was collected, washed with H_2_O and Et_2_O and dried under vacuum.

##### *fac*-[Re(5dmb)(CO)_3_-(*η* ^1^-ph)](CF_3_SO_3_^–^) (**Re(5)-ph**)

Starting from *fac*-Re(5dmb)(CO)_3_Br (0.21 g, 0.4 mmol) and following the general strategy, the target **Re(5)-ph** was obtained as a pale solid (0.18 g, 42%). ^1^H-NMR (400 MHz, CD_3_CN): *δ* 8.30 (s, 2H, 5dmb-6,6′), 8.03 (d, 2H, *J* = 8.4 Hz, 5dmb-3,3′), 7.79 (dd, 2H, *J* = 8.4, 1.6 Hz, 5dmb-4,4′), 7.69–7.09 (m, 24H, P*Ph*_2_–C_6_ *H*_4_–P*Ph*_2_), 2.19 (s, 6H, 5dmb–C*H*_3_) ppm. ^31^P-NMR (161 MHz): *δ* 18.9 (Re–*P*Ph_2_–C_6_H_4_–), –6.3 (Re–PPh_2_–C_6_H_4_–*P*Ph_2_) ppm. FT-IR (CH_3_CN): *ν*(CO) 2040, 1955, 1924 cm^–1^. ESI-MS (CH_3_CN): *m*/*z* 901 [M – PF_6_]^+^.

##### *fac*-[Re(tmb)(CO)_3_-(*η* ^1^-ph)](CF_3_SO_3_^–^) (**Re(4·5)-ph**)

Starting from *fac*-Re(tmb)(CO)_3_Br (0.33 g, 0.59 mmol) and following the general strategy, the target **Re(4·5)-ph** was obtained as a pale solid (0.27 g, 43%). ^1^H-NMR (400 MHz, CD_3_CN): *δ* 8.16 (s, 2H, tmb-3,3′), 7.94 (s, 2H, tmb-6,6′), 7.45–7.09 (m, 24H, P*Ph*_2_–C_6_ *H*_4_–P*Ph*_2_), 2.35 (s, 6H, tmb–C*H*_3_–4,4′), 2.10 (s, 6H, tmb–C*H*_3_–5,5′) ppm. ^31^P-NMR (161 MHz): *δ* 18.2 (Re–*P*Ph_2_–C_6_H_4_–), –6.3 (Re–PPh_2_–C_6_H_4_–*P*Ph_2_) ppm. FT-IR (CH_3_CN): *ν*(CO) 2038, 1950, 1921 cm^–1^. ESI-MS (CH_3_CN): *m*/*z* 929 [M – PF_6_]^+^.

##### *fac*-[Re(dmxb)(CO)_3_-(*η* ^1^-ph)](CF_3_SO_3_^–^) (**Re(OMe)-ph**)

Starting from *fac*-Re(dmxb)(CO)_3_Br (0.36 g, 0.63 mmol) and following the general strategy, the target **Re(OMe)-ph** was obtained as an off-white solid (0.33 g, 49%). ^1^H-NMR (400 MHz, CD_3_CN): *δ* 8.02 (d, 2H, *J* = 9.2 Hz, dmxb-3,3′), 7.98 (d, 2H, *J* = 2.8 Hz, dmxb-6,6′), 7.51 (dd, 2H, *J* = 9.2, 2.8 Hz, dmxb-4,4′), 7.47–7.09 (m, 24H, P*Ph*_2_–C_6_ *H*_4_–P*Ph*_2_), 3.79 (s, 6H, dmxb–OC*H*_3_) ppm. ^31^P-NMR (161 MHz): *δ* 19.3 (Re–*P*Ph_2_–C_6_H_4_–), –6.2 (Re–PPh_2_–C_6_H_4_–*P*Ph_2_) ppm. FT-IR (CH_3_CN): *ν*(CO) 2041, 1955, 1923 cm^–1^. ESI-MS (CH_3_CN): *m*/*z* 933 [M – PF_6_]^+^.

##### Synthesis of [Re(4dmb)(CO)_3_(*η* ^2^-ph)Re(4dmb)(CO)_2_(*η* ^2^-ph)Re(4dmb)(CO)_3_](PF_6_)_3_ (**L*3*(4)**)

**L*2*(4)** (OTf^–^ salt) (0.2 g, 0.12 mmol) was irradiated for 20 min in degassed CH_2_Cl_2_ (250 mL). After evaporation of the solvent, the crude red solid was dissolved together with **Re(4)-ph** (OTf^–^ salt) (0.15 g, 0.14 mmol) in THF (50 mL) and the mixture was heated under reflux under dim light for 24 h. The solvent was evaporated and the crude residue was purified using SEC. The fraction containing **L*3*(4)** was collected, evaporated and portioned between CH_2_Cl_2_ and NH_4_PF_6_ aqueous solution. The organic layer was washed once more with aqueous NH_4_PF_6_ solution, dried over Na_2_SO_4_ and evaporated to afford 0.21 g of yellow solid (65%). ^1^H-NMR (400 MHz, CD_3_COCD_3_): *δ* 8.55 (d, 4H, *J* = 5.6 Hz, 4dmb_ex_-6,6′), 8.35 (s, 4H, 4dmb_ex_-3,3′), 8.11 (s, 2H, 4dmb_in_-3,3′), 7.75 (d, 2H, *J* = 5.6 Hz, 4dmb_in_-6,6′), 7.53–7.13 (m, 52H, 4 × P*Ph*_2_ + P–C_6_ *H*_4_–P + 4dmb_ex_-5,5′), 6.54 (d, 2H, *J* = 5.6 Hz, 4dmb_in_-5,5′), 2.47 (s, 12H, 4dmb_ex_–C*H*_3_), 2.27 (s, 6H, 4dmb_in_–C*H*_3_) ppm. ^31^P-NMR (161 MHz): *δ* 23.7 (P_ex_–C_6_H_4_–*P*_in_), 19.6 (*P*_ex_–C_6_H_4_–P_in_). ESI-MS (CH_3_CN): *m*/*z* 743 [M – 3PF_6_]^3+^.

##### Synthesis of [{Re(4dmb)(CO)_2_(*η* ^2^-ph)}_3_](PF_6_)_3_ (**R(4)**) (Scheme S1, ESI†)^17^

A degassed solution of **L*3*(4)** (210 mg, 0.08 mmol) in an acetone–H_2_O mixture (290 mL, 7 : 1 v/v) was irradiated until the starting compound was consumed, and both terminal CO ligands were substituted by solvent (3 h), as monitored using ESI-MS (*m*/*z* 752 [M – 3PF_6_]^3+^). The mixture was evaporated, and the residue was dissolved in CH_3_CN and evaporated to dryness (this procedure was repeated three times). The crude **L*3*(4)–(CH_3_CN)_2_** was dissolved together with *p*-bis(diphenylphosphino)benzene (40 mg, 0.09 mmol) in acetone (40 mL) and refluxed under Ar in dim light for 24 h. Afterwards, the reaction mixture was evaporated and the crude material was purified using SEC. The fraction containing **R(4)** was collected, evaporated and portioned between CH_2_Cl_2_ and NH_4_PF_6_ aqueous solution. The organic layer was washed once more with aqueous NH_4_PF_6_ solution, dried over Na_2_SO_4_ and evaporated. After short column chromatography (SiO_2_, CH_3_CN–CH_2_Cl_2_ 1 : 5), a final recrystallization from EtOH–CH_2_Cl_2_ yielded **R(4)** as dark yellow crystals (132 mg, 54%). ^1^H-NMR (400 MHz, CD_3_CN): *δ* 8.20 (s, 6H, 4dmb-3,3′), 8.06 (d, 6H, *J* = 5.6 Hz, 4dmb-6,6′), 7.83 m, 12H, (P–C_6_ *H*_4_–P), 7.34 (t, 12H, *J* = 7.6 Hz, Ph-*p*), 7.26 (t, 24H, *J* = 7.6 Hz, Ph-*m*), 7.08 (m, 24H, Ph-*o*), 6.98 (d, 6H, *J* = 5.6 Hz, 4dmb-5,5′), 2.45 (s, 18H, 4dmb–C*H*_3_) ppm. ^31^P-NMR (161 MHz): *δ* 20.5 (*P*Ph_2_–C_6_H_4_–*P*Ph_2_) ppm. FT-IR (CH_3_CN): *ν*(CO) 1936, 1877, 1864 (sh) cm^–1^. ESI-MS (CH_3_CN): *m*/*z* 873 [M – 3PF_6_]^3+^. Anal. calcd (%) for C_132_H_108_F_18_N_6_O_6_P_9_Re_3_: C, 51.92; H, 3.56; N, 2.75; found: C, 51.81; H, 3.58; N, 2.79.

#### General procedure for the synthesis of **R(X)** (Scheme 1)

An acetone solution containing **Re(x)-ph** and Me_3_NO (1.05 eq.) was stirred at rt for 1 h. The temperature was gradually increased and the mixture was refluxed under Ar in dim light for 2 d. The solvent was evaporated and the mixture was portioned between CH_2_Cl_2_ and H_2_O. The organic layer was washed twice with H_2_O to remove Me_3_N^+^, dried over Na_2_SO_4_ and evaporated. Further purification was analogous to that for **R(4)**.

##### [{Re(5dmb)(CO)_2_(*η* ^2^-ph)}_3_](PF_6_)_3_ (**R(5)**)

Starting from 180 mg of **Re(5)-ph** (0.17 mmol) and following the general strategy, the target **R(5)** was obtained as yellow crystals (33 mg, 19%). ^1^H-NMR (400 MHz, CD_3_CN): *δ* 8.33 (br s, 12H, P–C_6_ *H*_4_–P), 7.90 (d, 6H, *J* = 9.2 Hz, 5dmb-3,3′), 7.52–7.47 (m, 12H, 5dmb-4,4′ + 5dmb-6,6′), 7.21 (t, 12H, *J* = 7.6 Hz, Ph-*p*), 7.11 (t, 24H, *J* = 7.6 Hz, Ph-*m*), 6.96 (m, 24H, Ph-*o*), 1.85 (s, 18H, 5dmb–C*H*_3_) ppm. ^31^P-NMR (161 MHz): *δ* 23.3 (*P*Ph_2_–C_6_H_4_–*P*PPh_2_) ppm. FT-IR (CH_3_CN): *ν*(CO) 1938, 1880, 1867 (sh) cm^–1^. ESI-MS (CH_3_CN): *m*/*z* 873 [M – 3PF_6_]^3+^. Anal. calcd (%) for C_132_H_108_F_18_N_6_O_6_P_9_Re_3_: C, 51.92; H, 3.56; N, 2.75; found: C, 51.85; H, 3.51; N, 2.77.

##### [{Re(tmb)(CO)_2_(*η* ^2^-ph)}_3_](PF_6_)_3_ (**R(4·5)**)

Starting from 250 mg of **Re(4·5)-ph** (0.23 mmol) and following the general strategy, the target **R(4·5)** was obtained as yellow crystals (45 mg, 19%). ^1^H-NMR (400 MHz, CD_3_CN): *δ* 8.32 (br s, 12H, P–C_6_ *H*_4_–P), 7.81 (s, 6H, tmb-3,3′), 7.36 (s, 6H, tmb-6,6′), 7.20 (t, 12H, *J* = 7.5 Hz, Ph-*p*), 7.09 (t, 24H, *J* = 7.6 Hz, Ph-*m*), 6.97 (m, 24H, Ph-*o*), 2.24 (s, 18H, tmb–C*H*_3_–4,4′), 1.76 (s, 18H, tmb–C*H*_3_-5,5′) ppm. ^31^P-NMR (161 MHz): *δ* 22.9 (*P*Ph_2_–C_6_H_4_–*P*Ph_2_) ppm. FT-IR (CH_3_CN): *ν*(CO) 1935, 1877 cm^–1^. ESI-MS (CH_3_CN): *m*/*z* 901 [M – 3PF_6_]^3+^. HRMS (ESI-TOFMS) (CH_3_CN): *m*/*z* [M – 3PF_6_]^3+^ calcd for C_138_H_120_N_6_O_6_P_6_Re_3_: 900.8794. Found: 900.8712.

##### [{Re(dmxb)(CO)_2_(*η* ^2^-ph)}_3_](PF_6_)_3_ (**R(OMe)**)

Starting from 300 mg of **Re(OMe)-ph** (0.28 mmol) and following the general strategy, the target **R(OMe)** was obtained as light yellow crystals (61 mg, 21%). ^1^H-NMR (400 MHz, CD_3_CN): *δ* 8.33 (br s, 12H, P–C_6_ *H*_4_–P), 7.90 (d, 6H, *J* = 9.2 Hz, dmxb-3,3′), 7.26–7.19 (m, 18H, dmxb-4,4′ + Ph-*p*), 7.16–7.09 (m, 30H, dmxb-6,6′ + Ph-*m*), 6.97 (m, 24H, Ph-*o*), 3.57 (s, 18H, dmxb–OC*H*_3_) ppm. ^31^P-NMR (161 MHz): *δ* 23.7 (*P*Ph_2_–C_6_H_4_–*P*PPh_2_) ppm. FT-IR (CH_3_CN): *ν*(CO) 1938, 1880 cm^–1^. ESI-MS (CH_3_CN): *m*/*z* 905 [M – 3PF_6_]^3+^. Anal. calcd (%) for C_132_H_108_F_18_N_6_O_12_P_9_Re_3_: C, 50.34; H, 3.46; N, 2.67; found: C, 50.35; H, 3.50; N, 2.68.

## Conclusions

We developed a new synthesis route for Re(i)-diimine trinuclear cyclic complexes, which exhibit highly suitable photophysical and electrochemical properties as redox PSs, *i.e.*, strong absorption in the visible region, high oxidation power and a long lifetime of the excited state, good stability and a strong reduction power of the reduced form. Employing these PSs in a visible-light-driven CO_2_ reduction in tandem with several mononuclear metal-complex catalysts even at equimolar concentrations demonstrated excellent photocatalytic efficiencies. High product selectivity (CO or HCOOH) with very high *Φ*_CO_ (up to 0.74) using a Re(i) catalyst and the highest *Φ*_HCOOH_ (0.58 using the Ru(ii) catalyst and 0.48 using the Mn(i) catalyst), even when employing triethanolamine as the electron donor without any other stronger electron donor, such as BIH or BI(OH)H. These excellent efficiencies and high stabilities during the photocatalytic reactions strongly suggest that the Re-rings have great potential for use in other redox photocatalytic systems.

## Note added after first publication

This article replaces the version published on 20th July 2016, in which some of the authors' corrections were omitted through editorial error.
