# Supplementary material for: Rhenium(i) trinuclear rings as highly efficient redox photosensitizers for photocatalytic CO2 reduction
Source: Chem Sci. 2016 Jul 5;7(11):6728–39. doi: 10.1039/c6sc01913g (PMC5355825; doi:10.1039/c6sc01913g)
Supplement: Supplementary file 1 [file SC-007-C6SC01913G-s001.pdf]

Electronic Supplementary Material (ESI) for Chemical Science.
This journal is © The Royal Society of Chemistry 2016

Supporting Information

Rhenium(I) Trinuclear Rings as Highly Efficient Redox Photosensitizers for Photocatalytic CO_2 Reduction

Jana Rohacova and Osamu Ishitani*

Department of Chemistry, Graduate School of Science and Engineering, Tokyo Institute of Technology, 2-12-1-NE-1 Ookayama, Meguro-ku, Tokyo, 152-8550, Japan

*Corresponding author: ishitani@chem.titech.ac.jp

Table of Contents

1. Supporting Scheme (S1)
2. Supporting Equations (S1–S2)
3. Supporting Tables (S1–S5)
4. Supporting Figures (S1–S19)
5. Supporting References

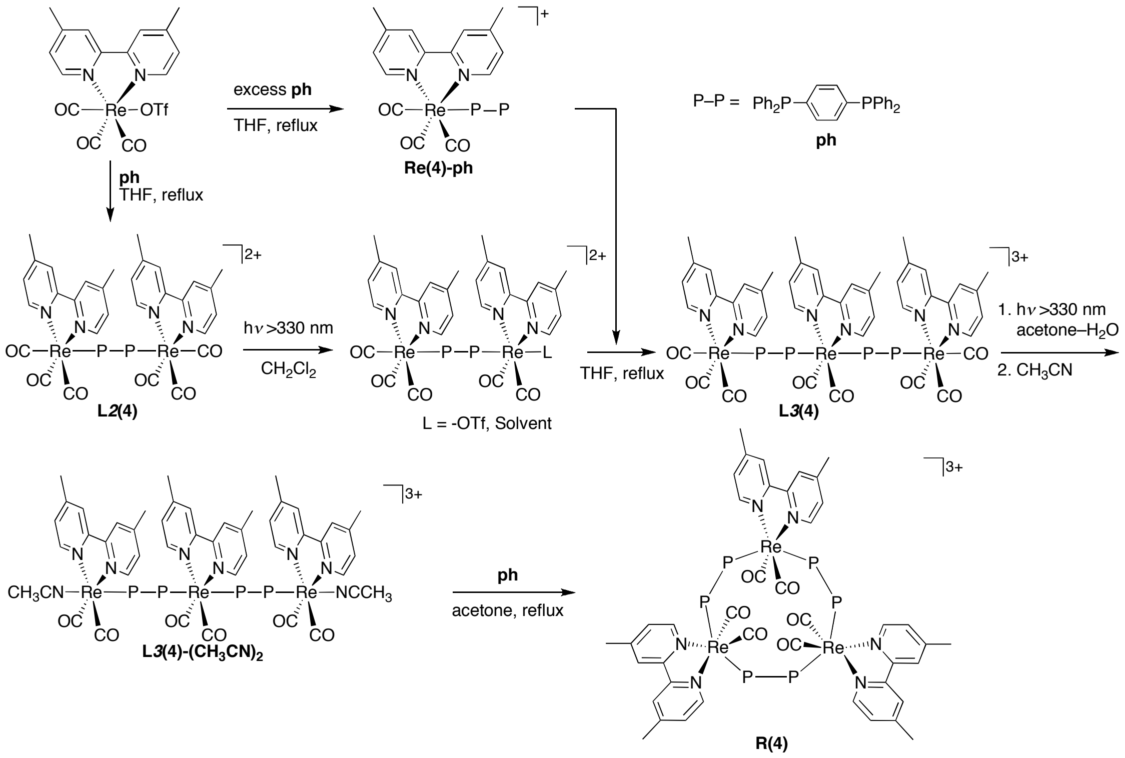

Scheme S1. Multistep synthetic approach of **R(4)** using photochemical ligand-substitution reaction.

Franck–Condon Analysis

The nonvibrational, room temperature emission spectra of **R(X)** were analysed by single-mode Franck–Condon analysis (eqn (S1)).[S1,S2] The photon numbers of the emission spectrum were corrected in a wavenumber scale using the equation $I(\nu) = I(\lambda) \times \lambda^2$, *i.e.*, $I(\nu)$ is the emission intensity at the energy in wavenumber (cm^{-1}) relative to that of the emitted intensity at the maximum (0 → 0 transition). E_0 is the energy gap between the zeroth vibrational levels in the ground and excited states, $\Delta\nu_{1/2}$ is the full width at half-maximum (fwhm) for an individual vibronic line and S_M is the corresponding electron-vibrational coupling constant or Huang–Rhys factor. The quantum spacing for the averaged medium-frequency vibrational mode, $\hbar\omega_M$, was fixed at *ca.* 1300 cm^{-1} or *ca.* 1700 cm^{-1} in the fits. The parameter υ_M is the vibrational quantum number for the medium frequency acceptor mode, and the summation was carried out over 6 vibrational levels: 0 → 5.

The fitted E_0 and $\Delta\nu_{1/2}$ values may be used to calculate the free energy of the MLCT excited state, ΔG^0_{MLCT}, according to eqn (S2) and are listed in Table S1.

$$I(\nu) = \sum_{\upsilon_M=0}^{\infty} \left[\frac{E_0 - \upsilon_M \hbar\omega_M}{E_0}\right]^4 \left[\frac{S_M^{\upsilon_M}}{\upsilon_M!}\right] \exp\left\{-4\ln 2\left[\frac{\nu - E_0 + \upsilon_M \hbar\omega_M}{\Delta\nu_{1/2}}\right]^2\right\} \quad \text{(S1)}$$

$$\Delta G^0_{MLCT} = E_0 + \frac{(\Delta\nu_{1/2})^2}{16\, k_B T \ln 2} \quad \text{(S2)}$$

Table S1. Energy of the MLCT excited state of **R(X)** at 25 ºC

	Solvent	$\Delta G^0_{\mathrm{MLCT}}$ / eV
**R(4)**	DMF	2.49
**R(5)**	DMF	2.57
	DMA	2.59
**R(4·5)**	DMF	2.65
	DMA	2.67
**R(OMe)**	DMF	2.52
**R(5)-e**	DMF	2.50

Table S2. Properties of **R(4·5)** in DMA at 25 ºC

	$\lambda_{\mathrm{abs}}{}^{a}$ / nm (ε / M^{-1} cm^{-1})	$\lambda_{\mathrm{em}}{}^{b}$ / nm	$\Phi_{\mathrm{em}}{}^{b}$	$\tau_{\mathrm{em}}{}^{c}$ / μs	$E_{1/2}{}^{\mathrm{red}\ d}$ / V	$E_{\mathrm{red}}{}^{*e}$ / eV	$K_{\mathrm{SV}}{}^{f}$ / M^{-1}	$k_q{}^{g}$ / M^{-1} s^{-1}	$\eta_q{}^{h}$
**R(4·5)**	385 (12100)	542	0.53	3.15	−1.84	0.83	9.0	2.8×10^6	0.92

[a] MLCT band. [b] λ_{ex} = 400 nm. [c] λ_{ex} = 401 nm. [d] First reduction potential *vs.* Ag/$AgNO_3$, determined from the DPV peak. [e] $E_{\mathrm{red}}* \approx \Delta G^0_{\mathrm{MLCT}} + E_{\mathrm{red}}$. [f] Stern–Volmer constant obtained from quenching experiment of emission by TEOA. [g] $k_q = K_{\mathrm{SV}}/\tau_{\mathrm{em}}$. [h] [TEOA] = 1.256 M.

Table S3. Absorption and electrochemical properties of the catalysts measured at 25 ºC

	$\lambda_{\mathrm{abs}}{}^{a}$ / nm (ε / M^{-1} cm^{-1})	ε_{436} (ε_{405}) / M^{-1} cm^{-1}	$E_p{}^{\mathrm{red}\ b}$ / V
**Re-ACN**[c]	353 (3600)	490 (1650)	−1.68
**Ru(*t*Bu)-Cl_2**[d]	349 (2000)	50	−1.57
**Mn(*t*Bu)-ACN**[d]	389 (3400)	1450	−1.68

[a] MLCT band. [b] Irreversible wave (the reduction potential *vs.* Ag/$AgNO_3$ was determined from the DPV peaks.
[c] Measured in a CO_2-saturated DMF–TEOA (5 : 1 v/v). [d] Measured in a CO_2-saturated DMA–TEOA (5 : 1 v/v).

Table S4. Fraction of the absorbed light by **R(X)** in regard to **Re-ACN** at different wavelengths (in DMF)

	λ / nm (**R(X)** : **Re-ACN** molar ratio)	
	436 (1 : 1)	405 (2 : 1)
**R(4)**	90%	89%
**R(5)**	87%	88%
**R(4·5)**	85%	89%
**R(OMe)**	75%	88%

Table S5. Photocatalytic CO_2 reduction using **R(4·5)** photosensitizer with various catalysts[a]

	TON			Φ^{b}
	HCOOH	CO	H_2	HCOOH
**Ru(*t*Bu)-Cl_2**[c]	50	15	15	0.45
**Mn(*t*Bu)-ACN**[d]	18	9	traces	0.18[e]

[a] Photocatalytic CO_2 reduction using an DMA–TEOA mixture (5 : 1 v/v) containing **R(4·5)** (0.05 mM) as a PS and various catalysts (0.05 mM) under 436 nm light irradiation of 4.2×10^{-9} einstein s^{-1} intensity. [b] ± 2%; [c] TON at 16 h of irradiation. [d] TON at 12 h of irradiation. [e] Taking into account absorption of the Mn-dimer.

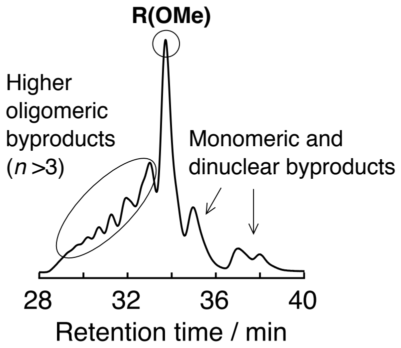

Fig. S1. Size exclusion chromatogram of the crude reaction mixture of **R(OMe)** synthesis according to Scheme 1 (main text).

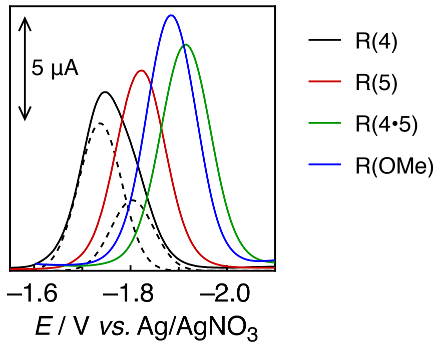

Fig. S2. Differential pulse voltammograms of the first reduction of **R(X)** (0.5 mM) in DMF under Ar. The DPV peak of **R(4)** was deconvoluted with two Gaussian functions (---).

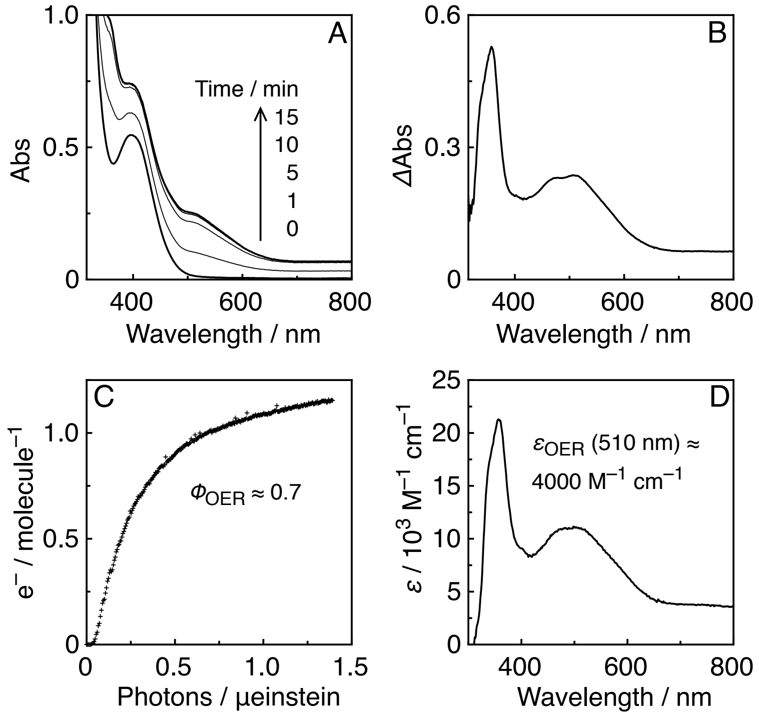

Fig. S3. (A) UV−vis absorption spectral changes of an Ar-saturated DMF−TEOA (5 : 1 v/v) solution containing **R(4)** (0.05 mM) under irradiation at λ_{ex} = 436 nm (5.6 × 10^{-9} einstein s^{-1}). (B) Differential absorption spectra

derived from the spectrum before irradiation and after 15 min of irradiation. (C) Time course of accumulated electrons in **R(4)** as function of absorbed photons within 10 min of irradiation, compensated for the inner filter effect. (D) Differential absorption spectrum of **R(4)** before and after bulk electrolysis in an Ar-saturated DMF solution at –1.95 V *vs.* Ag/$AgNO_3$.

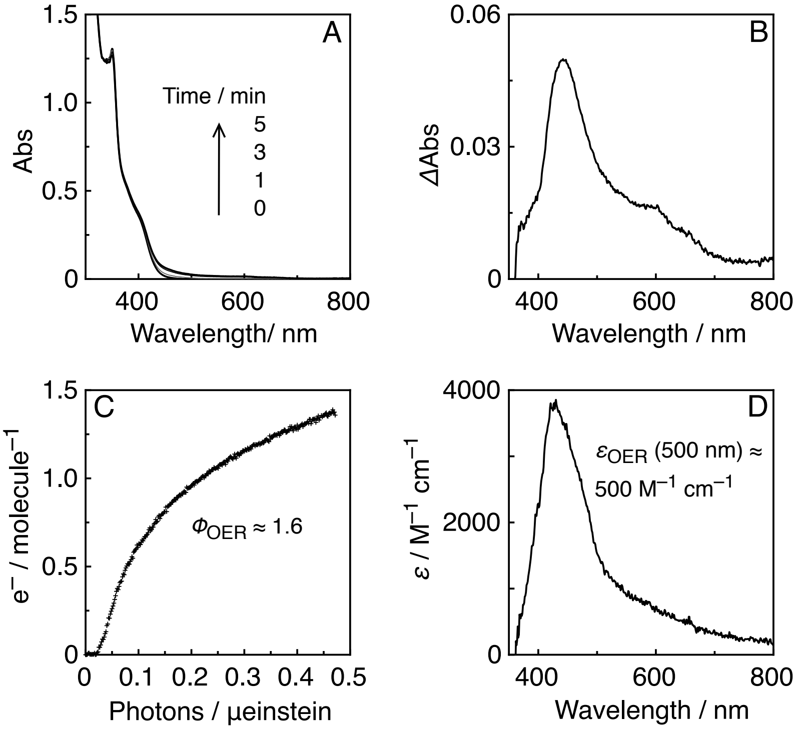

Fig. S4. (A) UV−vis absorption spectral changes of an Ar-saturated DMF−TEOA (5 : 1 v/v) solution containing **R(OMe)** (0.05 mM) under irradiation at λ_{ex} = 405 nm (1.3 × 10^{-9} einstein s^{-1}). (B) Differential absorption spectra derived from the spectrum before irradiation and after 5 min of irradiation. (C) Time course of accumulated electrons in **R(OMe)** as function of absorbed photons within 10 min of irradiation, compensated for the inner filter effect. (D) Differential absorption spectrum of **R(OMe)** before and after bulk electrolysis in an Ar-saturated DMF solution at –2.0 V *vs.* Ag/$AgNO_3$.

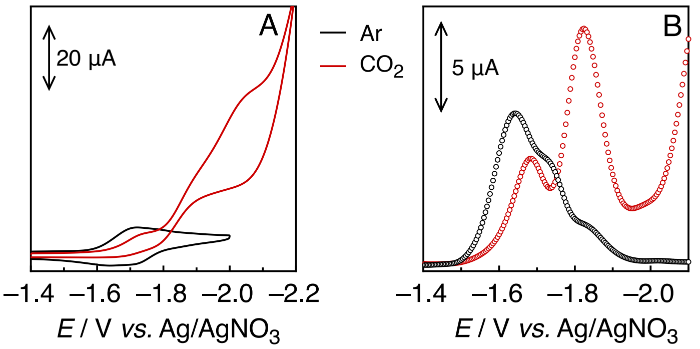

Fig. S5. Cyclic voltammogram (A) and corresponding differential pulse voltammogram (B) of **Re-Solv** (0.5 mM) in DMF–TEOA (5 : 1 v/v), where Solv = TEOA under Ar (black) or Solv = CO_2-TEOA under CO_2 (red) atmosphere, respectively; CV sweep rate = 100 mV s^{-1}.

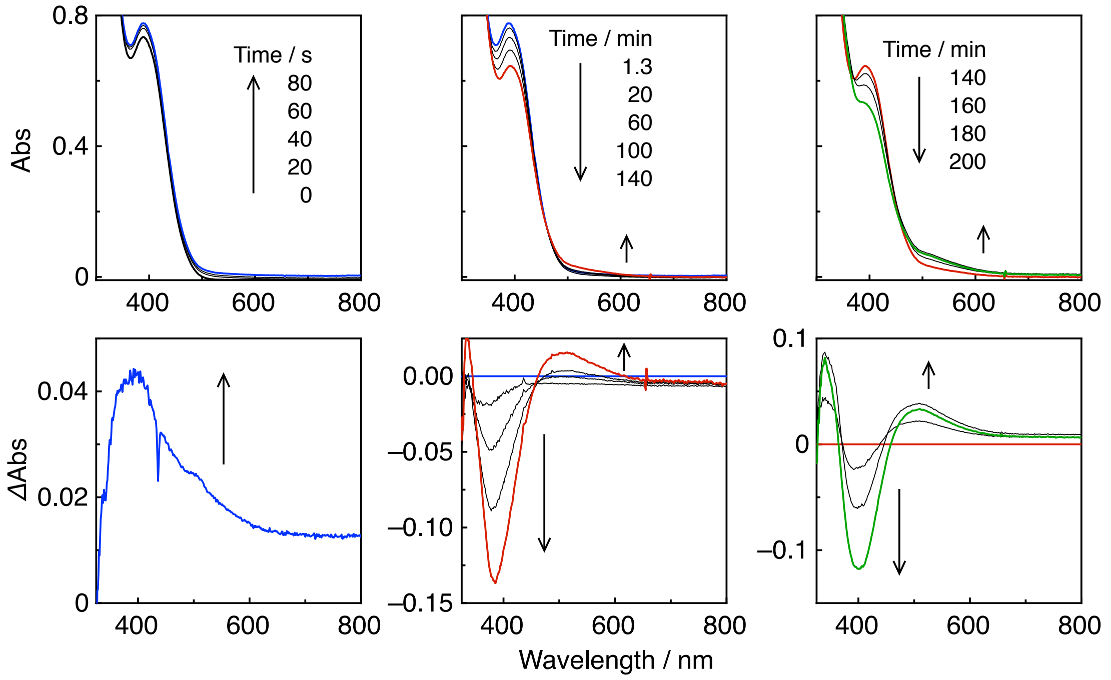

Fig. S6. UV−vis (top) and corresponding differential (bottom) absorption spectral changes of a CO_2-saturated DMF−TEOA (5 : 1 v/v) solution containing **R(4)** (0.05 mM) as a PS and **Re-ACN** (0.05 mM) as the catalyst, under irradiation at λ_{ex} = 436 nm (5.7 × 10^{-9} einstein s^{-1}) in the initial stage (left) and over 3.5 h of irradiation (middle and right).

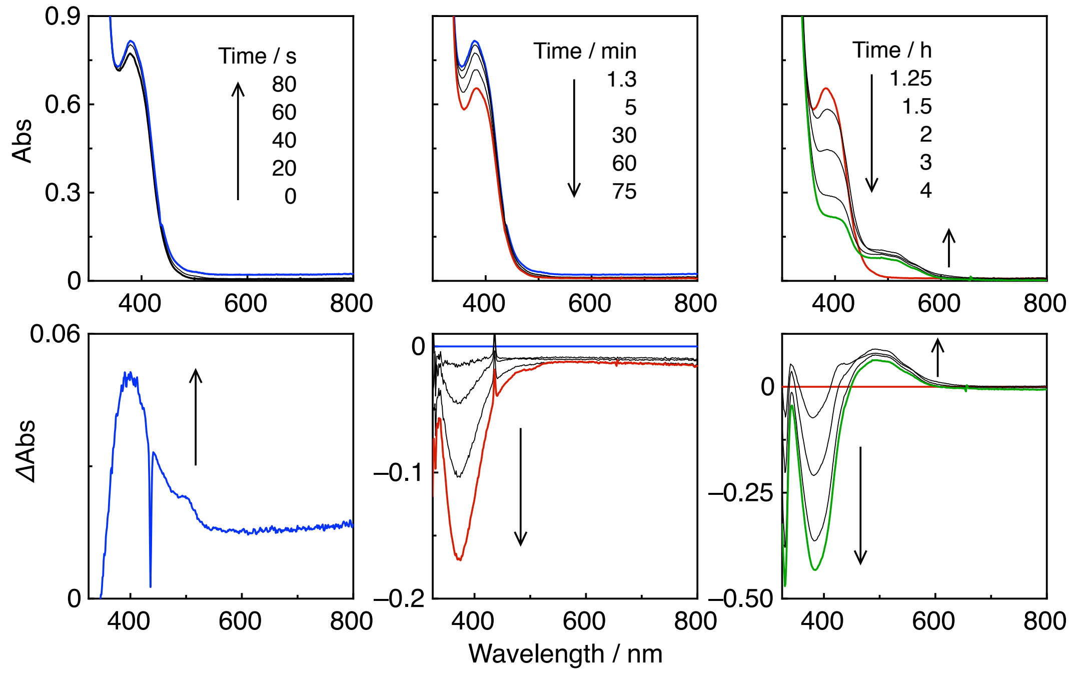

Fig. S7. UV−vis (top) and corresponding differential (bottom) absorption spectral changes of a CO_2-saturated DMF−TEOA (5 : 1 v/v) solution containing **R(4·5)** (0.05 mM) as a PS and **Re-ACN** (0.05 mM) as the catalyst, under irradiation at λ_{ex} = 436 nm (5.7 × 10^{-9} einstein s^{-1}) in the initial stage (left) and over 4 h of irradiation (middle and right).

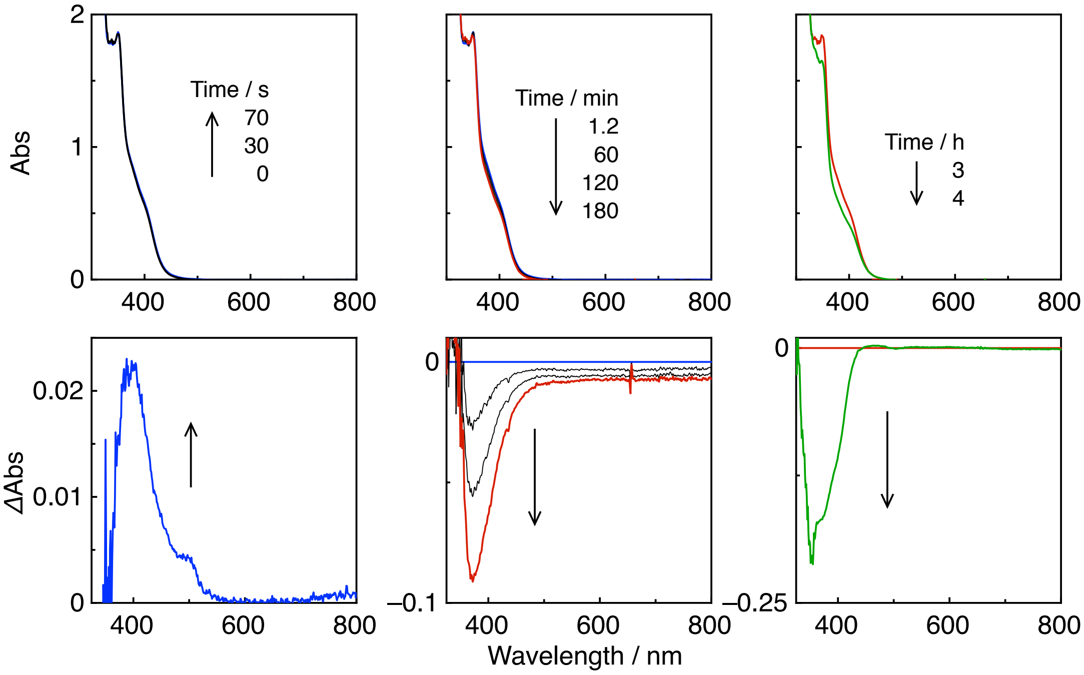

Fig. S8. UV−vis (top) and corresponding differential (bottom) absorption spectral changes of a CO_2-saturated DMF−TEOA (5 : 1 v/v) solution containing **R(OMe)** (0.05 mM) as a PS and **Re-ACN** (0.05 mM) as the catalyst, under irradiation at λ_{ex} = 436 nm (5.7 × 10^{-9} einstein s^{-1}) in the initial stage (left) and over 4 h of irradiation (middle and right).

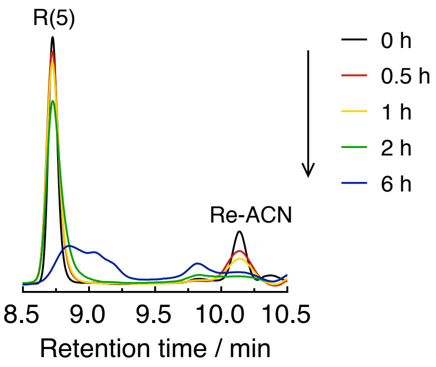

Fig. S9. UPLC chart of the **R(5)**/**Re-ACN** photocatalytic system ; λ_{det} = 350 nm.

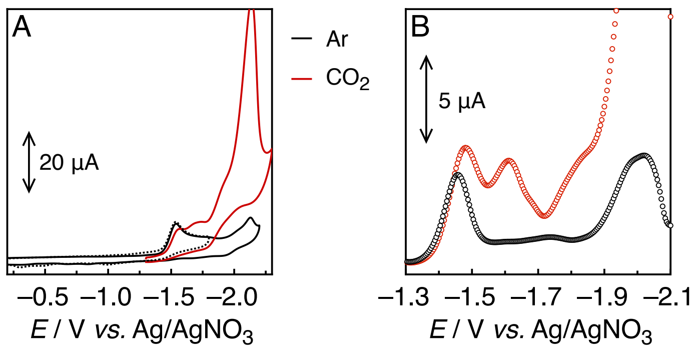

Fig. S10. Cyclic voltammogram (A) and corresponding differential pulse voltammogram (B) of **Ru(*t*Bu)-Cl₂** (0.5 mM) in DMA–TEOA (5 : 1 v/v) under Ar (black) or CO_2 (red) atmosphere; CV sweep rate = 100 mV s^{-1}.

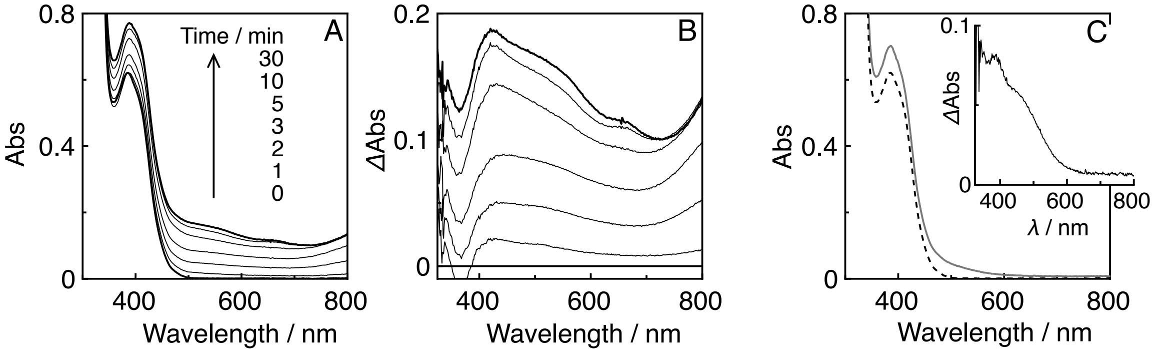

Fig. S11. UV−vis (A) and corresponding differential (B) absorption spectral changes of an Ar-saturated DMA−TEOA (5 : 1 v/v) solution containing **R(5)** (0.05 mM) and **Ru(*t*Bu)-Cl$_2$** (0.05 mM), over 1 h irradiation at λ_{ex} = 436 nm (4.2 × 10^{-9} einstein s^{-1}). (C) UV–vis spectra of the solution before irradiation (black dashed), and that of the irradiated solution after exposure to the air (gray solid); Inset: Corresponding differential UV–vis spectrum.

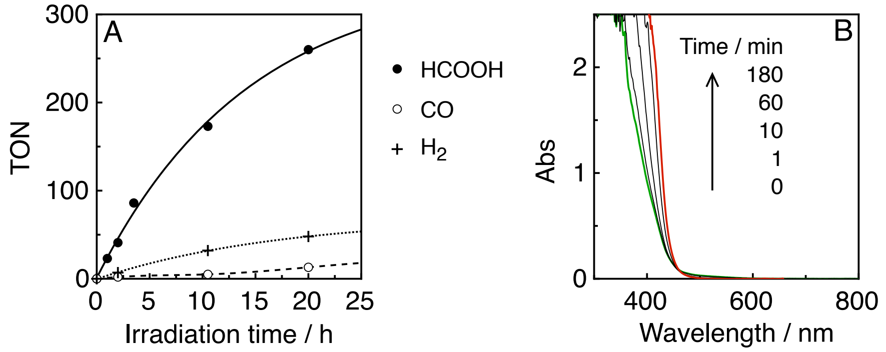

Fig. S12. (A) Products-time course of photocatalytic CO_2 reduction using **R(5)** (0.05 mM) as a PS, **Ru(*t*Bu)-Cl$_2$** (0.05 mM) as the catalysts and BI(OH)H (0.03M) as a sacrificial electron donor in DMA–TEOA (5 : 1 v/v) under 436-nm light irradiation of 4.2 × 10^{-9} einstein s^{-1} intensity. (B) UV–vis absorption spectral changes of the irradiated solution.

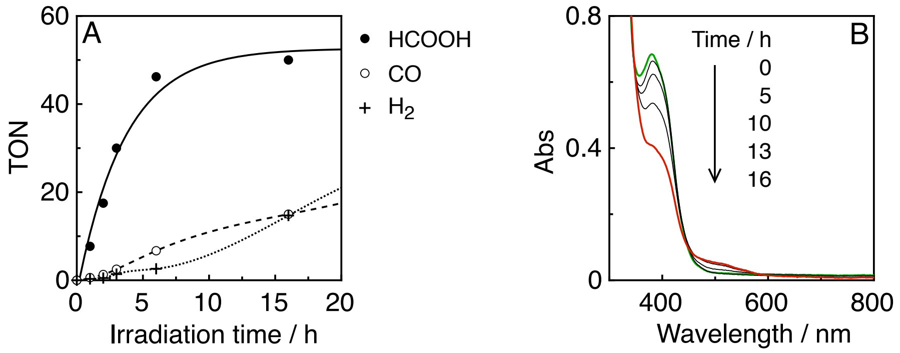

Fig. S13. (A) Products-time course of photocatalytic CO_2 reduction using **R(4·5)** (0.05 mM) as a PS and **Ru(*t*Bu)-Cl$_2$** (0.05 mM) as the catalysts in DMA–TEOA (5 : 1 v/v) under 436-nm light irradiation of 4.2 × 10^{-9} einstein s^{-1} intensity. (B) UV–vis absorption spectral changes of the irradiated solution.

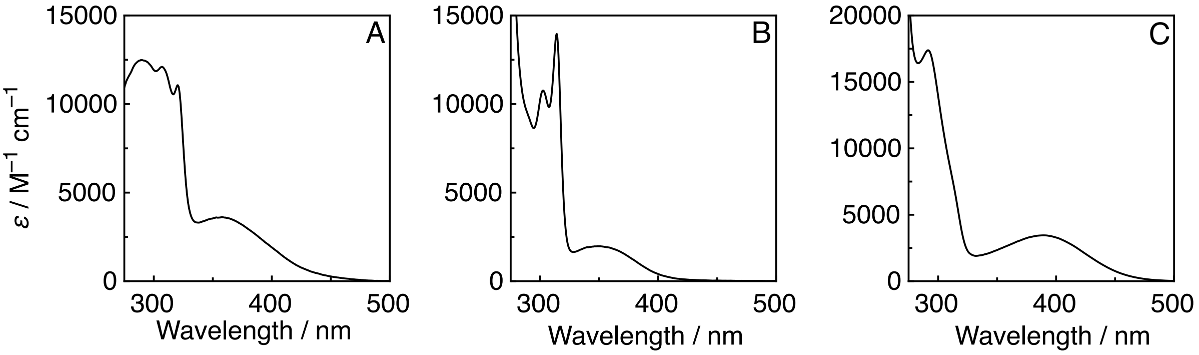

Fig. S14. (A) UV–vis absorption spectrum of **Re-ACN** (A) in a CO_2-saturated DMF–TEOA (5 : 1 v/v), **Ru(*t*Bu)-Cl$_2$** (B) and **Mn(*t*Bu)-ACN** (C) both in a CO_2-saturated DMA–TEOA (5 : 1 v/v).

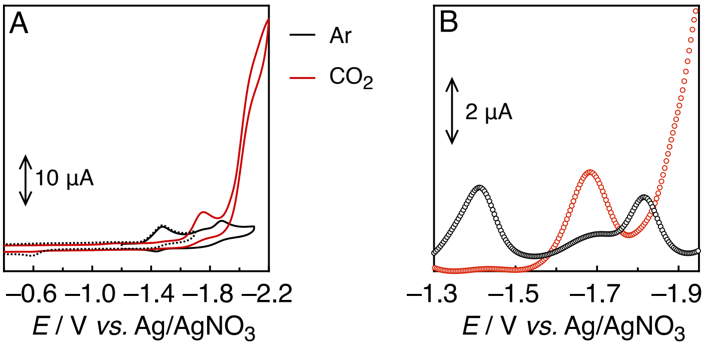

Fig. S15. Cyclic voltammogram (A) and corresponding differential pulse voltammogram (B) of **Mn(*t*Bu)-Solv** (0.5 mM) in DMA–TEOA (5 : 1 v/v), where Solv = TEOA under Ar (black) or Solv = CO_2-TEOA under CO_2 (red) atmosphere, respectively; CV sweep rate = 100 mV s^{-1}.

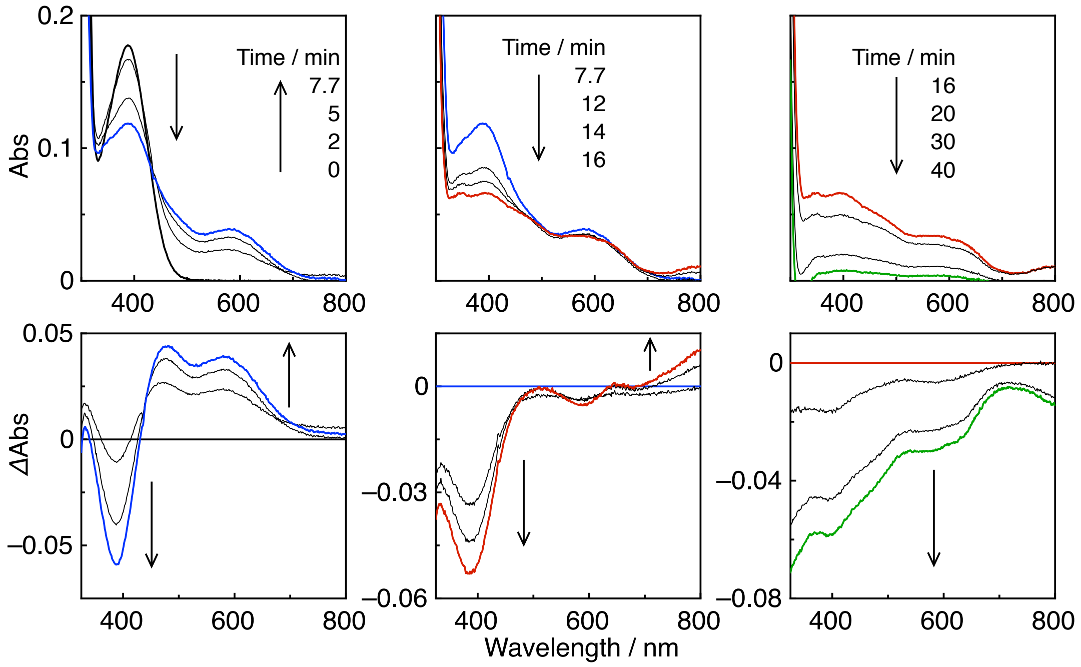

Fig. S16. UV−vis (top) and corresponding differential (bottom) absorption spectral changes of a CO_2-saturated DMA−TEOA (5 : 1 v/v) solution containing **Mn(*t*Bu)-ACN** (0.05 mM) as photocatalyst under irradiation at λ_{ex} = 436 nm over 1 h irradiation.

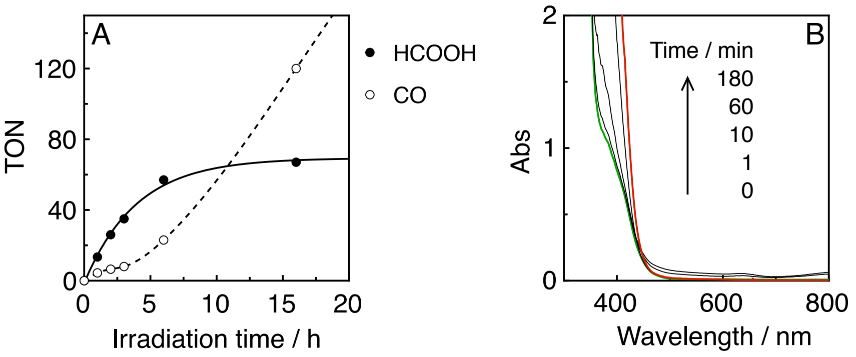

Fig. S17. (A) Products-time course of photocatalytic CO_2 reduction using **R(5)** (0.05 mM) as a PS, **Mn(*t*Bu)-ACN** (0.05 mM) as the catalysts and BI(OH)H (0.03M) as a sacrificial electron donor in DMA–TEOA (5 : 1 v/v) under 436-nm light irradiation of 5.3 × 10^{-9} einstein s^{-1} intensity. (B) UV–vis absorption spectral changes of the irradiated solution.

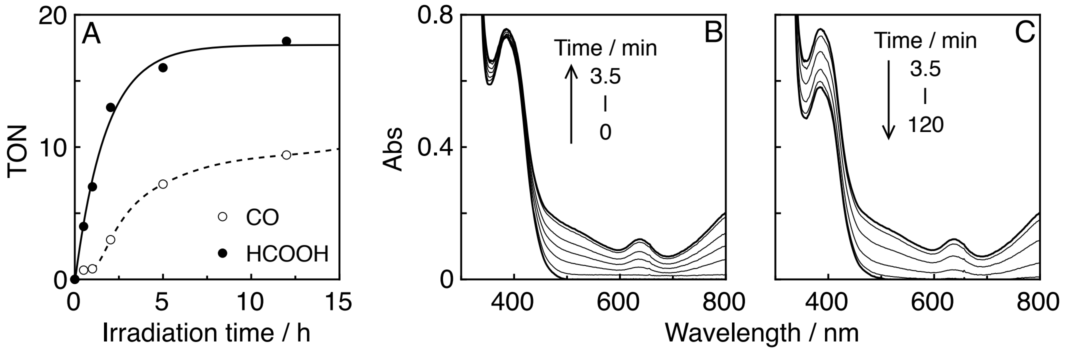

Fig. S18. (A) Time course of product formation using **R(4·5)** (0.05 mM) as a PS and **Mn(*t*Bu)-ACN** (0.05 mM) as the catalysts in DMA–TEOA (5 : 1 v/v) under 436 nm-light irradiation of 4.2 × 10^{-9} einstein s^{-1} intensity. UV−vis absorption spectral changes of the photocatalytic reaction in the initial stage (B) and over 2 h irradiation (C).

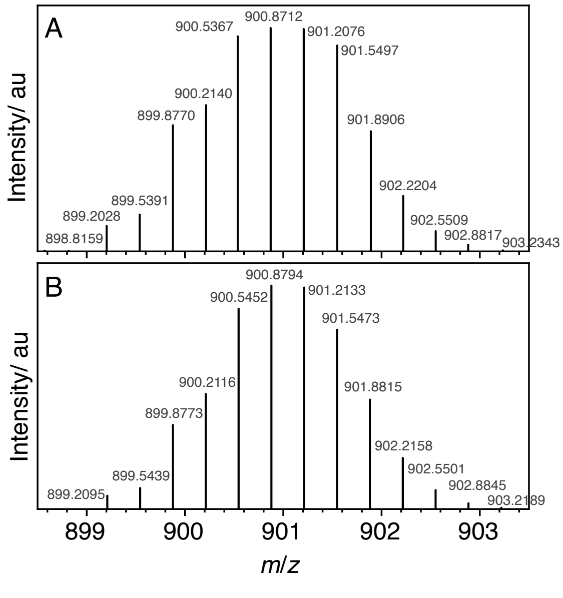

Fig. S19. Peaks corresponding to $[M - 3PF_6]^{3+}$ in the ESI-TOFMS spectrum of **R(4·5)** (A) and the calculated isotope distribution pattern (B).

Supporting References

S1 J. V. Caspar, T. D. Westmoreland, G. H. Allen, P. G. Bradley, T. J. Meyer and W. H. Woodruff, *J. Am. Chem. Soc.*, 1984, **106**, 3492–3500.

S2 L. A. Worl, R. Duesing, P. Chen, L. Della Ciana and T. J. Meyer, *J. Chem. Soc., Dalton Trans.*, 1991, 849–858.
